# What the Phage: a scalable workflow for the identification and analysis of phage sequences

**DOI:** 10.1093/gigascience/giac110

**Published:** 2022-11-18

**Authors:** Mike Marquet, Martin Hölzer, Mathias W Pletz, Adrian Viehweger, Oliwia Makarewicz, Ralf Ehricht, Christian Brandt

**Affiliations:** Institute of Infectious Diseases and Infection Control, Jena-University Hospital/Friedrich Schiller University, Jena 07747, Germany; Center of Sepsis Control and Care (CSCC), Jena 07747, Germany; Leibniz Center for Photonics in Infection Research (LPI), Jena 07747, Germany; Bioinformatics and Systems Biology, Robert Koch Institute, Berlin 13353, Germany; Institute of Infectious Diseases and Infection Control, Jena-University Hospital/Friedrich Schiller University, Jena 07747, Germany; Center of Sepsis Control and Care (CSCC), Jena 07747, Germany; Leibniz Center for Photonics in Infection Research (LPI), Jena 07747, Germany; InfectoGnostics Research Campus, Jena 07747, Germany; Medical Microbiology and Virology, University Hospital Leipzig, Leipzig 04103, Germany; Institute of Infectious Diseases and Infection Control, Jena-University Hospital/Friedrich Schiller University, Jena 07747, Germany; Center of Sepsis Control and Care (CSCC), Jena 07747, Germany; Leibniz Center for Photonics in Infection Research (LPI), Jena 07747, Germany; InfectoGnostics Research Campus, Jena 07747, Germany; InfectoGnostics Research Campus, Jena 07747, Germany; Optisch-molekulare Diagnostik und Systemtechnologie, Leibniz Institute of Photonic Technology (Leibniz-IPHT), Jena 07747, Germany; Institute of Physical Chemistry, Friedrich-Schiller-University Jena, Jena 07747, Germany; Institute of Infectious Diseases and Infection Control, Jena-University Hospital/Friedrich Schiller University, Jena 07747, Germany; Leibniz Center for Photonics in Infection Research (LPI), Jena 07747, Germany; InfectoGnostics Research Campus, Jena 07747, Germany

**Keywords:** phage prediction, easy to use, Nextflow, Docker, multitool approach, scalable

## Abstract

Phages are among the most abundant and diverse biological entities on earth. Phage prediction from sequence data is a crucial first step to understanding their impact on the environment. A variety of bacteriophage prediction tools have been developed over the years. They differ in algorithmic approach, results, and ease of use. We, therefore, developed “What the Phage” (WtP), an easy-to-use and parallel multitool approach for phage prediction combined with an annotation and classification downstream strategy, thus supporting the user's decision-making process by summarizing the results of the different prediction tools in charts and tables. WtP is reproducible and scales to thousands of datasets through a workflow manager (Nextflow). WtP is freely available under a GPL-3.0 license (https://github.com/replikation/What_the_Phage).

## Background

Bacteriophages (phages) are viruses that infect prokaryotes and replicate by utilizing the host's metabolism [1, 2]. They are among the most abundant and diverse organisms on the planet and inhabit almost every environment [2]. The double-stranded DNA–tailed phages possibly make up the majority of phages on the planet [3]. Single-stranded DNA, single-stranded RNA, and double-stranded RNA viruses are minor groups [4].

Phages drive and maintain bacterial diversity by perpetuating the coevolutionary interactions with their bacterial prey, facilitating horizontal gene transfer and nutrient turnover through continuous cycles of predation and coevolution [5, 6]. They directly impact the microbiome (e.g., the human gut) and can influence human health [7]. At the same time, phages in aquatic habitats are responsible for the mortality of nearly 20–40% of prokaryotes every day [8]. However, despite having considerable impacts on microbial ecosystems, they remain one of the least understood members of complex communities [9].

Sequencing the entire DNA of environmental samples (metagenomics) is an essential approach to gaining insights into the microbiome and functional properties.

It should be noted that due to the genome size of phages ranging from 5 to 500 kbp [10], their entire genome can be sequenced via long-read technologies (e.g., Oxford Nanopore Technologies or PacBio) [11]. These sequencing techniques facilitate phage genome recovery in their natural habitat without the need to culture their hosts to isolate the phages [2] and sequencing of soil or ocean samples on-site (e.g., with the portable MinION sequencing device). Such technological developments led to a rapid increase in human gut virome studies [12] and the discovery of novel, uncharacterized phages from environmental metagenomes [13, 14].

These advances demand reliable and easy-to-use phage prediction tools and workflows that can be directly used on assembled sequencing data. However, predicting phages from metagenomes and their differentiation from prophages remains a challenge as there is no established computational gold standard [13].

Existing prediction tools rely on direct comparison of sequence similarity [15, 16], sequence composition [17, 18], and models based on these features derived through learning algorithms [15,16, 19]. The phage prediction tool DeepVirFinder uses a *k*-mer–based deep learning method using convolutional neural networks and builds on its predecessor, VirFinder [18, 20]. PPR-Meta also utilizes convolutional neural networks to identify phages and plasmids [19]. Metaphinder integrates BLAST hits to multiple genomes in a database to identify phage sequences in assembled contigs [21]. Seeker and VirNet work with a deep learning framework that uses long short-term memory models that do not depend on sequence motives [22,23], while Vibrant utilizes deep learning neural networks based on protein signatures [15]. Virsorter2 builds on the strategy of Virsorter (first iteration) by applying machine learning to evaluate the viral content using genomic features [16, 24]. Phigaro uses precomputed sets of pVOG profile hidden Markov models (HMMs) [25].

The performance of each prediction method varies [26] depending on the sample type or material, the sequencing technology, and the assembly method, which makes the correct choice for any given sample difficult without having to install and test several tools.

The user can choose from many tools based on different calculation strategies, software dependencies, and databases to further complicate matters. We observed various installation issues and conflicts while working with these phage prediction tools, making a multitool screening approach complex and time-consuming.

To overcome these obstacles and issues, we developed “What the Phage” (WtP), a reproducible, accessible, and scalable workflow utilizing the advantages of multiple prediction tools in parallel to detect and annotate phages.

## Methods

### Design and implementation

**Figure 1:**
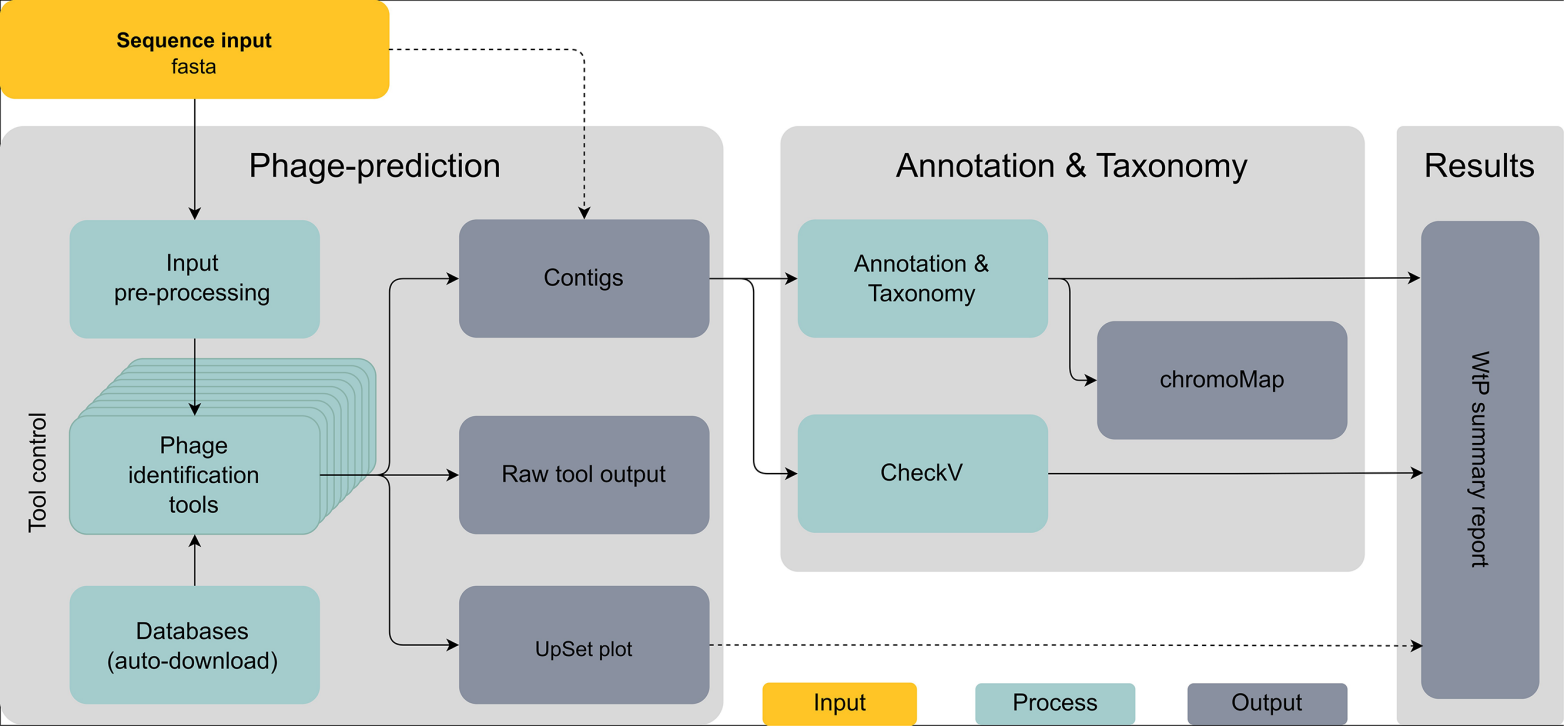
Simplified “What the Phage” workflow flowchart. Sequence input (yellow) can either be first run through the “prediction” and subsequently “Annotation & Taxonomy” as a whole or used directly as an input for the “Annotation & Taxonomy” only. Each of the multiple phage prediction tools can be individually controlled if needed (tool control).

WtP was implemented in Nextflow, a portable, scalable, and parallelizable workflow manager [27]. At the time of writing, 11 different tools to predict phage sequences and other annotation and classification programs are included in WtP. WtP uses so-called containers (Docker or Singularity [Apptainer]) for an installation-free workflow execution without dependency or operating system conflicts for each of the currently over 21 programs included. All containers are prebuilt, version controlled, online available at the dockerhub website, and automatically downloaded. Additionally, all 9 different databases (belonging to the corresponding tools) and datasets used by the workflow are managed automatically. The modular code structure and functionalities of Nextflow and Docker/Singularity (Apptainer) allow easy integration of other phage prediction tools and additional analysis steps in future releases of the pipeline. The workflow consists of 2 main phases, which are executed subsequently or, if specified, individually (Fig. 1):

1. Prediction: The prediction of putative phage sequences
2. Annotation & Taxonomy: The gene annotation and taxonomic classification of phage sequences

### Prediction and visualization

**Figure 2:**
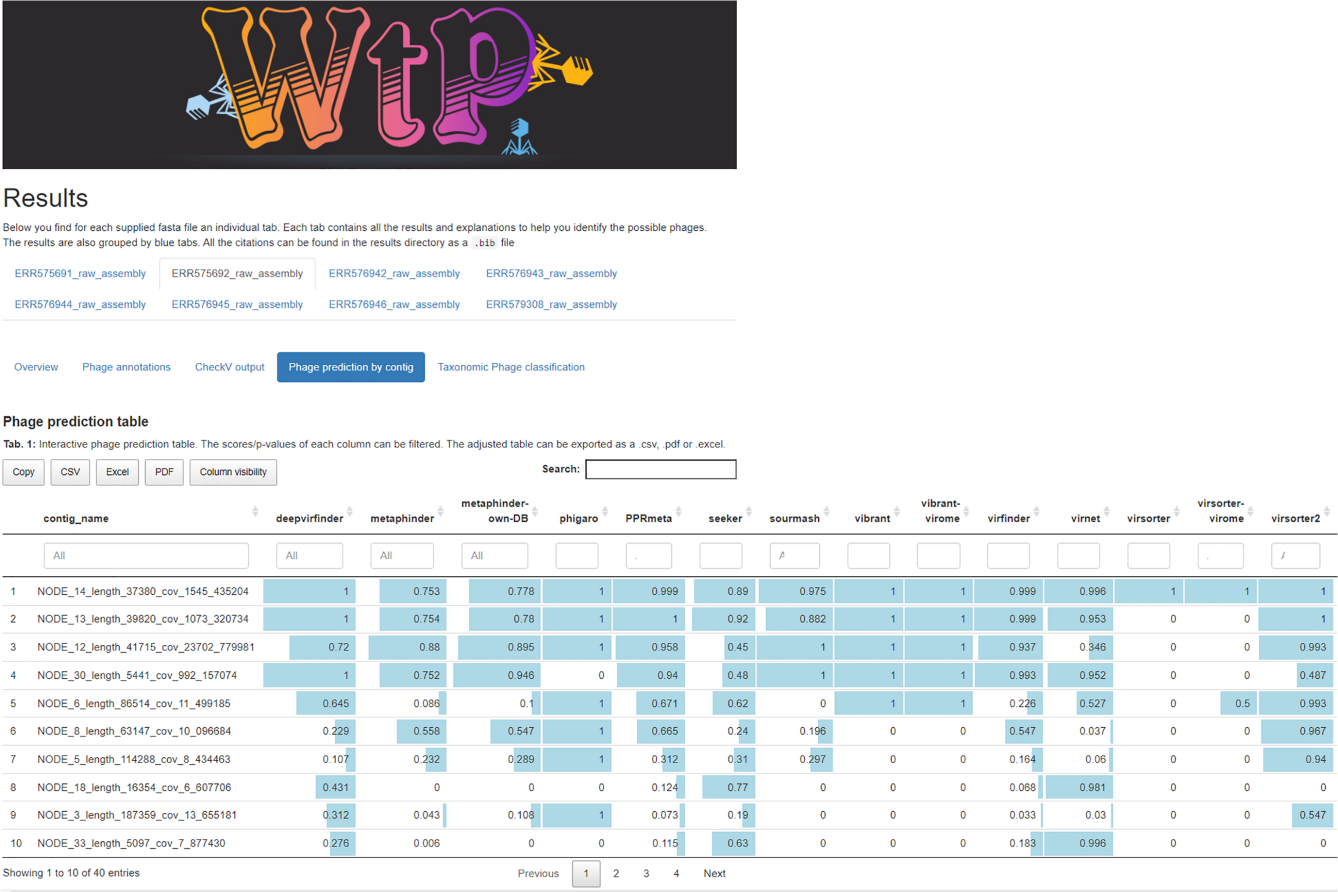
Example figure of the “Phage prediction by contig table” section of the result report. The “Phage prediction by contig table” section summarizes the tool outputs for the analyzed sample ERR575692. The full result report can be found in the Data Availability section [30]. All tables can be exported as Excel, PDF, or CSV files by using the buttons above the tables.

The first stage takes a multi-fasta file as input (e.g., a metagenomic assembled contigs), formats it to the demands of each tool, and filters sequences below a user-defined length threshold (1,500 bp by default) via SeqKit v0.10.1 (RRID:SCR_018926) [28]. Sequences that are too small usually generate false-positive hits, as Gregory et al. [29] observed. The phage prediction process is performed by 11 different tools (14 approaches) in parallel: VirFinder v1.1 [18], PPR-Meta v1.1 (RRID:SCR_016915) [19], VirSorter v1.0.6 (with and without virome mode) [16], DeepVirFinder v1.0 [20], Metaphinder with no release version (using default database and own database (Zheng et al. database, GitHub commit ID bebc447d00ec9ff9f4960f38b627d8651262ff72) [21], sourmash v2.0.1 [17], Vibrant v1.2.1 (with and without virome mode) [15], VirNet v0.1 [23], Phigaro v2.2.6 [25], Virsorter2 v2.0 [24], and Seeker [22] with no release version (GitHub commit ID 9ae14887dcd4295f4340626d06d8848cead2102d). Tool outputs are collected in a detailed result report (see Result Report section, Fig. 2; Data Availability section [30]).

### Functional annotation & taxonomy

For this step, Prodigal v2.6.3–1 (RRID:SCR_011936) [31] is used in metagenome mode to predict open reading frame (ORFs) and HMMER v3.3 (Default cutoff: -E 1e-30; RRID:SCR_005305) [32] to identify homologs via the pVOG database [33]. All annotations are summarized in an interactive HTML file via chromoMap [34] (see Fig. 4). Additionally, WtP classifies all contigs via sourmash and provides a probability score to the corresponding taxonomic classification based on the Zheng et al. database [35].

### Result report

WtP streamlines the detection of phage sequences across multiple tools in their default settings, thus balancing some drawbacks (e.g., relying on updated databases, only predicting phages available in databases). To ease the data interpretation for the user, WtP collects the results in a detailed summary report HTML file for simplified interpretation (Fig. 2, Data Availability section [30]). The report contains an UpSet plot summarizing the prediction performance of each tool (Fig. 2). Finally, the “phage prediction by contig table” (Fig. 2) summarizes tool outputs for each contig. WtP assigns numeric values to tools that do not generate *P* values or scores between 0 and 1 (see Result Report, “phage prediction by contig section”) and sorts them based on phage likelihood. All the results are individually filterable so that the user can consider additional insights or information provided by community platforms such as IMG/VR [36] or iVirus [37].

### Other features

All mandatory databases and containers are automatically downloaded when the workflow is started and stored for the following executions. Additionally, the workflow can be pre-setup to analyze sequences offline subsequently. WtP provides the raw output of each tool to support a transparent and reproducible mode of operation. Maximum execution stability is ensured by automatically excluding phage prediction tools that cannot analyze the input data without failing the workflow (e.g., file too large, not the scope of an individual tool). We also provide a detailed user manual that is regularly updated [38].

### Dependencies and version control

WtP requires the workflow management software Nextflow [27] and either Docker [39] or Singularity (Apptainer) [40] installed and configured on the system. The pipeline was tested on Ubuntu 16.04 LTS, Ubuntu 18.04 LTS, and Windows 10 (via Windows Subsystem for Linux 2 using Docker). The installation process is described in detail in the WtP user manual [38]. Each workflow release specifies the Nextflow version to avoid any version conflicts between the workflow code and the workflow manager. A specific Nextflow version can be directly downloaded as an executable file from the Nextflow website.

Additionally, each container used in the workflow is tagged by the accompanying tool version, prebuilt, and stored on hub.docker.com.

### Data description

To demonstrate the utility and performance of WtP, we analyzed a described metagenome data set (ENA Study PRJEB6941, ERR575692) using a local desktop machine (24 threads, 60 GB RAM, Ubuntu 18.04.4 LTS) and WtP release v1.1.0. Kleiner et al. [41] generated an artificial microbiome via bacteria and phage cultures in mice feces (germ-free C57BL/6 J mice) and sequenced the sample. The group added 6 phages (P22, T3, T7, ɸ6, M13, and ɸVPE25) and 2 bacteria (*Listeria monocytogenes and Bacteroides thetaiotaomicron*) to germ-free feces. We, therefore, expect the prediction of the 6 known phages and possibly other phage sequences related to both bacteria strains. Still, false-positive hits and tool disagreements are plausible results during the phage prediction process. The dataset analyzed in this study (ERR575692) is derived from Illumina HiSeq data.

### Analysis

**Figure 3:**
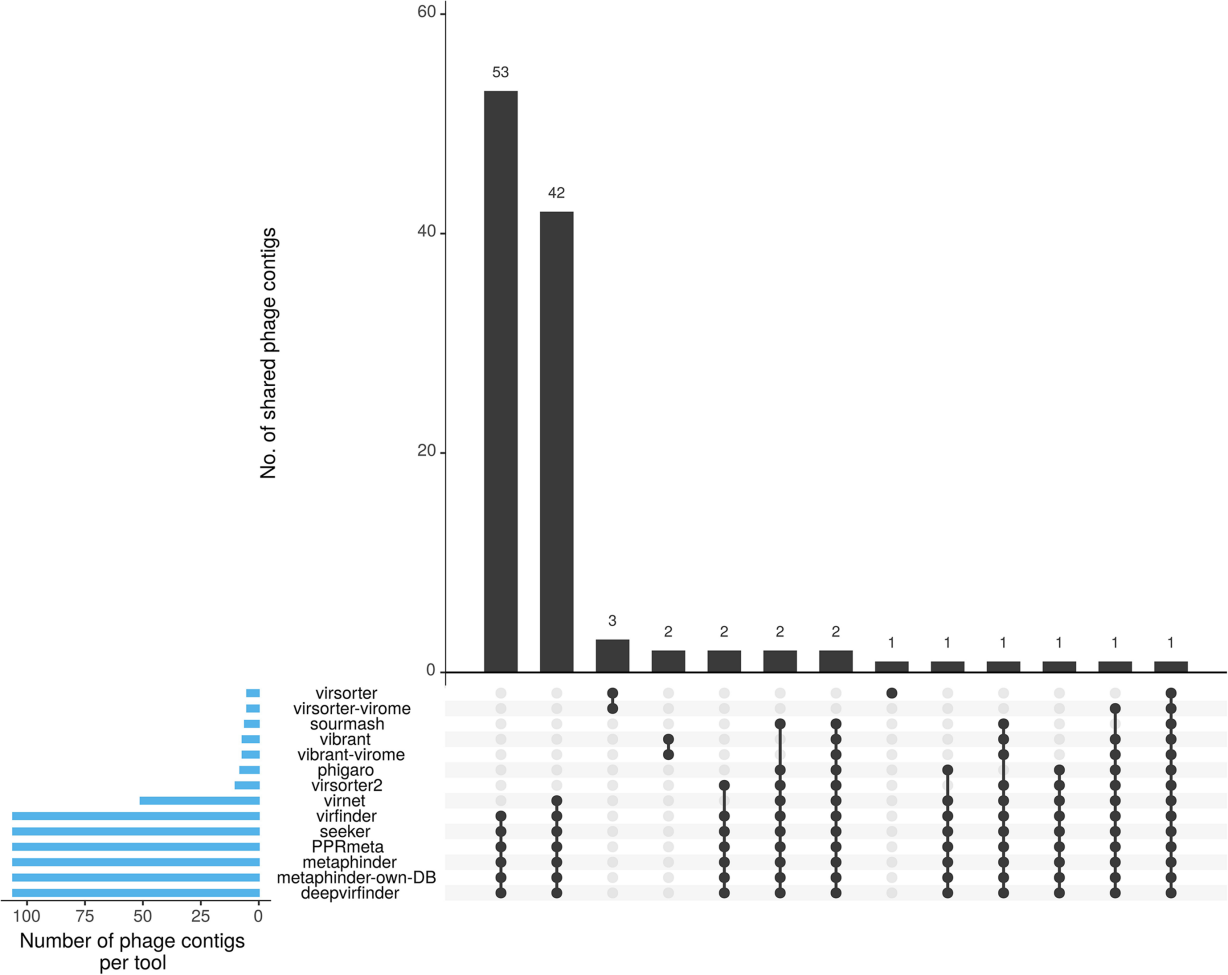
UpSet plot summarizing the prediction performance of each tool for the sample ERR575692. The total amount of identified phage contigs per tool is shown in blue bars on the left. Black, vertical bars visualize the number of contigs that each tool or tool combination has uniquely identified. Each tool combination is shown below the vertical barplot as a dot matrix. How to read the diagram: for example, 53 phage contigs are found by 6 tools (DeepVirFinder, Metaphinder-own-DB, Metaphinder, PPRmeta, Seeker, and VirFinder). Another 42 contigs are found by these tools but also Virnet.

The raw read data sets composed of 8 samples were downloaded from the ENA server and individually assembled via metaSPAdes v3.14 using the default settings [42]. The resulting assembly files, stored in the *GigaScience* GigaDB database [43], were analyzed with WtP (release v1.1.0, default settings). As WtP uses multiple tools for phage prediction, an UpSet plot summarizes for each sample the performance of all approaches executed successfully (Fig. 3 for sample ERR575692).

The complete result report for sample ERR575692 can be found in the Data Availability section [30].

**Figure 4:**
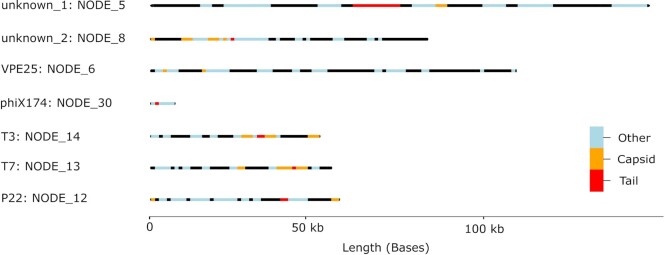
Visual annotation of phage contigs and annotated protein-coding genes via chromoMap. Annotations are colored based on the categories of capsid genes (orange), tail genes (red), and other genes (blue). Other contigs without either capsid or tail genes have been removed from this figure for better readability.

In general, the prediction values (*P* values, scores, and outputs generated by the phage prediction tools) were >0.7 for the first 4 sequences/contigs (NODE_14, NODE_13, NODE_12, NODE_30), indicating high consensus among the prediction tools, although in some cases, tool prediction values were below 0.5 (Phigaro: NODE_30, Seeker: NODE_12 and NODE_30, Virnet: NODE_12 and Virsorter2: NODE_30). Prediction values for NODE_6 were below 0.67, and Virsorter2 and Phigaro showed high values >0.99. The same applied to NODE_8 and NODE_5, indicating dissonance for these 3 contigs. Surprisingly, Virsorter and Virsorter-virome only predicted the sequence, NODE_14, as a phage. In case of dissonance and when tools coincided, validation of contigs via phage annotations and CheckV [44] simplified further assessment. In the case of sample ERR575692, phage genes (like tail and capsid genes) were annotated on all 7 contigs (Fig. 4).

The workflow was able to detect contigs that corresponded to the phages P22 (NODE_12), T3 (NODE_14), and T7 (NODE_13). In addition, the phage for the internal Illumina control (phiX174: NODE_30) was also predicted. The M13 phage [41] could not be identified as it was not assembled via metaSPAdes due to the low read abundance and low coverages (below 0.55×, determined by Kleiner et al. [38]). The same applies to phage ɸ6, which was not detectable by Kleiner et al. [38]. However, VPE25 (NODE_6) was initially not taxonomically classified by WtP as it was not represented in the taxonomic database (Zheng et al. database) at this time; however, the corresponding contig was annotated with essential phage genes (Fig. 4). Therefore, the unclassified contig was analyzed manually via blastn (nr/nt database) and resulted in the genome sequence of VPE25 (PRJEB13004).

Furthermore, CheckV determined a phage completeness score of over 89% for all 7 contigs (Table 1). In addition to the phages mentioned above, 2 more large contigs with capsid and tail gene annotations indicate prophage(s) of *Salmonella enterica* (contig NODE_5 and NODE_8). Both contigs showed tail and capsid genes and were labeled as prophages via CheckV with estimated completeness of over 99.99%.

**Table 1:** Summary of the CheckV output for the sample ERR575692. All contigs with a completeness >89% and a length >5,000 bp are shown.

Phage name	Contig_id	Gene count	CheckV quality	Completeness, %	Contig length, bp
unknown1	NODE_5	107	Complete	100.0	114,288
unknown2	NODE_8	71	High quality	100.0	63,147
VPE25	NODE_6	137	High quality	99.99	86,514
phiX174	NODE_30	8	Medium quality	89.35	5,441
T3	NODE_14	43	High quality	93.34	37,380
T7	NODE_13	53	Complete	99.48	39,820
P22	NODE_12	67	Complete	100.0	41,715

These results were manually confirmed using NCBI's blastn (nr/nt database). The sequences matched with 100% identity to *S. enterica*(*Salmonella enterica strain FDAARGOS_768 chromosome, complete genome*) but not to prophage sequences. Furthermore, NODE_8 had 1.37 times the contig length of the matched reference (from CheckV), and NODE_5 had 3.24 times the contig length of the matched reference, which may have influenced the NCBI blastn search. *S. enterica* is known to host prophages [45]; therefore, the identified prophage sequences of CheckV are plausible results.

### Performance assessment

The WtP meta-workflow utilizes several phage identification tools and allows simple execution of a single tool or multiple tools at once. WtP does not favor or disadvantage any prediction tools based on their performance but collects each raw tool output in a user-friendly, easy-to-read result overview.

We did not additionally benchmark the tools integrated into our workflow because the performance of most of them has recently been benchmarked independently [26].

Ho et al. [26] tested Virfinder v1.0, MetaPhinder, PPR_Meta v1.1, Seeker v1.0.3, Virfinder v1.1, VirSorter v1.06, and VirSorter2 v2.2.3 and utilized near-identical commands, parameters, and databases across the benchmarked prediction tools as WtP. Another benchmark would thus only duplicate prior work.

Most tools performed well in the benchmarking of Ho et al. [26], detecting the majority of phage sequences while keeping false positives low. PPR-Meta and VirSorter2, which use 2 different machine learning methods, had the best performance across the tools.

However, *k*-mer tools outperformed reference similarity and gene-based tools when tested on positive and negative phage datasets. Tests with randomly shuffled phage sequences showed a clear difference between machine learning and conventional tools.

The performance of most tools dropped significantly when a real metagenomic dataset was used compared to the RefSeq benchmark. The *k*-mer prediction tools showed a smaller drop in F1 score based on their RefSeq benchmark compared to reference similarity/gene-based tools as described by Ho et al. [26].

The group also pointed out that the tools with machine/deep learning algorithms can detect novel phages. However, their performance may be unpredictable when exposed to novel data with features that differ from those used in the training sets [26].

Therefore, we believe that a combination of phage prediction algorithms (machine/deep learning and similarity/gene based) is a good compromise for unknown and novel datasets.

WtP deploys the benchmarked tools by default (WtP v1.2.0). If users wish to deploy other tools that were not benchmarked by Ho et al. [26], they can activate them easily.

While a sensical approach, multiple tools can be combined in the prediction step to yield an “ensemble” approach. To benchmark this approach, however, against individual tools is beyond the scope of this work, which aimed to facilitate the accessibility to phage prediction tools.

### Limitations

Some limitations must be noted. No specialized phage assembly strategy or any cleanup step was included during the assembly step. Therefore, some smaller mice host contigs (below 5,000 bp) produced false-positive hits. However, these contigs were distinguishable after the “Annotation & Taxonomy” step both in CheckV and due to the lack of typical genes related to, for example, capsid or tail proteins, showing the application of WtP also for contaminated datasets. WtP does not filter the output of phage prediction tools for prophages, although the CheckV output indicates if a contig could be a prophage.

Furthermore, WtP uses default database(s) or the original trained model(s) provided with each stand-alone prediction tool. We note that most casual users are unlikely to retrain these tools before their use.

Accurate gene prediction from the phage genome is still difficult [46]. This fact has affected both phage prediction and functional gene annotation in virology. New phage gene databases and algorithms could improve the quality of gene prediction in the future. We, therefore, implemented the function to provide, for example, more recent databases to improve gene annotation.

## Discussion and Potential Implications

With the rise of metagenomics and the application of machine learning principles for virus detection, several phage prediction tools have been released over the past few years. All these tools utilize a variety of prediction approaches, all with advantages and limitations [26]. The user's choice for using certain tools often depends strongly on their usability and accessibility and less on performance. While some tools already come with a packaging system such as Conda or a containerized environment, there exists no general framework for their execution database dependencies, and installation issues prevent many potential users from using certain tools. At least 1 multitool approach was implemented on a smaller scale by Gregory et al. [29] (comprising only VirFinder and VirSorter).

The overarching goal of WtP is to make phage prediction tools more accessible for a broader user spectrum and non-bioinformaticians, as culture-free sequencing has led to the rapid increase of phage studies [12]. WtP acts as an ideal, all-encompassing starting point for any given assembly and provides a searchable and filterable report of the analyzed data. The user is provided with sufficient processed data (such as tool performance comparisons, taxonomic assessments, and annotation maps) to work reliably with the predicted sequences and support the decision-making process if different prediction tools are not in agreement with each other.

The meta-tool WtP allows the user to deploy current state-of-the-art phage prediction tools very easily, all at once, or only a selection of tools. WtP does not favor or disadvantage any prediction tools based on their performance analyzed in the benchmarking work of Ho et al. [26]. It is still the user's task to select the most likely phage contigs, extract them from WtPs output, and use them for a more detailed and curated analysis.

Further information and guides are provided either via the report or the hosted manual. WtP streamlines the prediction of phage sequence recognition across multiple tools in a reproducible and scalable workflow to allow researchers to focus on their scientific questions instead of software implementations.

## Future Directions

WtP is a workflow project that will be improved and extended as the modular approach and containerization simplify the integration of new tools. The predictive scope of WtP can be extended to other viruses (such as RNA viruses) and prophages by including future tools specifically designed for such use cases and adjusting filter and annotation steps. The modular nature of the workflow using Docker and Nextflow allows the integration of new phage prediction tools by request of users, thus allowing WtP to keep up with the fast-developing field of bioinformatic tools. The versioning of WtP represents a well-functioning approach with tested and up-to-date versions of the workflow. Thus, the correct functioning of the workflow is always guaranteed and allows a reliable and fast prediction of phage sequences.

## Availability of Supporting Source Code and Requirements

Project name: What the Phage (WtP)

Project homepage: https://github.com/replikation/What_the_Phage

Programming language: Nextflow, Bash, Python, R

Other requirements: Ubuntu 18.04 LTS, Docker-version 20.10.12, Nextflow-version 21.10.6

License: GPL-3.0

RRID: SCR_022 871

## Data Availability

The WtP user manual [38] and Result Report are available in GitHub [30]. The WtP result data storage [47] and WtP databases are available in OSF [48]. Data used in this work are available in GitHub [49]. All supporting data and materials are available in the *GigaScience* GigaDB database [43].

## Abbreviations

BLAST: Basic Local Alignment Search Tool; bp: base pair; NCBI: The National Center for Biotechnology Information; WtP: What the Phage.

## Competing interests

The authors declare that they have no competing interests.

## Authors' Contributions

MM and CB provided conceptualization, design, and implementation; conducted the experiment; and created the figures. All authors actively participated in the writing and editing of the manuscript. All authors have read and agreed to the published version of the manuscript.

## Funding

This project was funded by the Federal Ministry of Education and Research (BMBF, Germany) in the framework of the Integrated Research and Treatment Centres program via the Center for Sepsis Control and Care (CSCC), Grand No. 01EO1502 (PI: MWP). MM was funded within this project. CB and RE were funded by a collaborative R&D project (BMBF) Grand No. 3GW0423B (PI: OM) and 3GW0423C (PI: RE). Funding for open-access charge: The Open Access Publishing program of the German Research Foundation (DFG) via the Thuringian University and State Library (ThULB).

## Supplementary Material

giac110_GIGA-D-22-00131_Original_Submission

giac110_GIGA-D-22-00131_Revision_1

giac110_Response_to_Reviewer_Comments_Original_Submission

giac110_Reviewer_1_Report_Original_Submission Satoshi Hiraoka -- 6/20/2022 Reviewed

giac110_Reviewer_1_Report_Revision_1 Satoshi Hiraoka -- 9/2/2022 Reviewed

giac110_Reviewer_2_Report_Original_Submission Huaiqiu Zhu -- 7/7/2022 Reviewed
