# Supplementary material for: What the Phage: a scalable workflow for the identification and analysis of phage sequences
Source: Gigascience. 2022 Nov 18;11:giac110. doi: 10.1093/gigascience/giac110 (PMC9673492; doi:10.1093/gigascience/giac110)
Supplement: giac110_GIGA-D-22-00131_Revision_1 [file giac110_giga-d-22-00131_revision_1.pdf]

GigaScience

What the Phage: A scalable workflow for the identification and analysis of phage sequences

--Manuscript Draft--

**Manuscript Number:**	GIGA-D-22-00131R1
**Full Title:**	What the Phage: A scalable workflow for the identification and analysis of phage sequences
**Article Type:**	Technical Note
**Funding Information:**	Bundesministerium für Bildung und Forschung (01EO1502) — Not applicable<br>Bundesministerium für Bildung und Forschung (13GW0423B) — Not applicable
**Abstract:**	Phages are among the most abundant and diverse biological entities on earth. Phage prediction from sequence data is a crucial first step to understanding their impact on the environment. A variety of bacteriophage prediction tools have been developed over the years. They differ in algorithmic approach, results, and ease of use. We, therefore, developed "What the Phage" (WtP), an easy-to-use and parallel multitool approach for phage prediction combined with an annotation and classification downstream strategy, thus, supporting the user's decision-making process by summarizing the results of the different prediction tools in charts and tables. WtP is reproducible and scales to thousands of datasets through a workflow manager (Nextflow). WtP is freely available under a GPL-3.0 license (https://github.com/replikation/What_the_Phage).
**Corresponding Author:**	Mike Marquet<br>Jena University Hospital<br>Jena, GERMANY
**Corresponding Author Secondary Information:**	
**Corresponding Author's Institution:**	Jena University Hospital
**Corresponding Author's Secondary Institution:**	
**First Author:**	Mike Marquet
**First Author Secondary Information:**	
**Order of Authors:**	Mike Marquet<br>Martin Hölzer<br>Mathias W Pletz<br>Adrian Viehweger<br>Oliwia Makarewicz<br>Ralf Ehricht<br>Christian Brandt
**Order of Authors Secondary Information:**	
**Response to Reviewers:**	Revision for "What the Phage: A scalable workflow for the identification and analysis of phage sequences"<br><br>Dear Editor and Reviewers,<br><br>First and foremost, we thank the reviewers and the editor for taking the time to look through our manuscript. The suggestions were excellent and meaningful. We

Powered by Editorial Manager® and ProduXion Manager® from Aries Systems Corporation

addressed all the comments, and below you will find our point-by-point response to the reviewer’s comments. We feel the manuscript has improved because of these changes.

Reviewer #1
In this manuscript, the authors developed a new tool, What the Phage (WtP), for comparison of the output from multiple bioinformatics tools to predict phage sequences from genomic or metagenomic datasets. The purpose of this study is some or less meaningful. As the authors described in the Introduction section, currently it is difficult to predict reliable viral genomes, especially from culture-independent metagenomic datasets precisely because of the lack of knowledge about viral genomes in current protein/genome databases. There are many bioinformatics tools already proposed and some of them are widely used in microbiology, however, the outputs from these tools are frequently varied and conflicted among them. However, there is no good integrative platform to compare the outputs. Here, the proposed tool easily generates well-summarized output derived from multiple tools, and thus, the tool might be facilitated the analysis of phage prediction in the field of
microbiology. Indeed, the authors conducted (only but) one case study using real phage genomes and reported reasonable performance. I feel the tool has some potential to contribute to the wide fields of viral genomics.

However, the user of this tool should keep in mind the fact that the tool just summarizes the output of multiple phage-prediction tools, meaning does not evaluate the reliability of the output, as described in the Discussion section. I feel thus the tool sometimes may lead to misunderstandings or make the users confuse rather than help them. It should emphasize that the majority decision among the multiple tools does not always bring the best result. The users may need further detailed analysis for the precise prediction of viral genome from metagenomes.

This is a good point. We extended and updated the manuscript with the key messages of the benchmarking article by Ho et al. (https://www.biorxiv.org/content/10.1101/2021.04.12.438782v2). WtP executes now by default only the tools that were used in the benchmark study:

“The WtP meta-workflow utilizes several phage identification tools and allows simple execution of a single tool or multiple tools at once. WtP does not favor or disadvantage any prediction tools based on their performance but collects each raw tool output in a user-friendly, easy-to-read result overview.
We did not additionally benchmark the tools integrated into our workflow because the performance of most of them has recently been benchmarked independently [26].
Ho et al. tested Virfinder v1.0, MetaPhinder, PPR_Meta v1.1, Seeker v1.0.3, Virfinder v1.1, VirSorter v1.06, VirSorter2 v2.2.3 and utilized near identical commands, parameters and databases across the benchmarked prediction tools as WtP. Another benchmark would thus only duplicate prior work.
Most tools performed well in the benchmarking of Ho et al., detecting the majority of phage sequences while keeping false positives low. PPR-Meta and VirSorter2, which use two different machine learning methods, had the best performance across the tools.
However, k-mer tools outperformed reference similarity and gene-based tools when tested on positive and negative phage datasets. Tests with randomly shuffled phage sequences showed a clear difference between machine learning and conventional tools.
The performance of most tools dropped significantly when a real metagenomic dataset was used compared to the RefSeq benchmark. The k-mer prediction tools showed a smaller drop in F1-score based on their RefSeq benchmark compared to reference similarity/gene-based tools as described by Ho et al. [26].
The group also pointed out that the tools with machine/deep learning algorithms can detect novel phages. However, their performance may be unpredictable when exposed to novel data with features that differ from those used in the training sets.

Powered by Editorial Manager® and ProduXion Manager® from Aries Systems Corporation

	Therefore, we believe that a combination of phage prediction algorithms (machine/deep learning and similarity/gene-based) is a good compromise for unknown and novel datasets.<br>WtP deploys the benchmarked tools by default. If users wish to deploy other tools that were not benchmarked by Ho et al., they can activate them easily.<br>While a sensical approach, multiple tools can be combined in the prediction step to yield an "ensemble" approach. To benchmark this approach, however, against individual tools is beyond the scope of this work, which aimed to facilitate the accessibility to phage prediction tools."<br><br>We agree with the reviewer that the results may confuse the user, and that is why we added information (WtP v1.2.0) to the result report , in the end, to help understand the data.<br><br>Also, I feel that, because the development of bioinformatics tools is quite rapid, integrated platforms like WtP will be outdated very soon without continuous effort for maintenance and upgrade to assimilate future novel tools. I understand the 'sustainability' of the tool is out of the journal scope, but the perspective on this point will be better to be described in the manuscript or GitHub page.<br><br><br>We understand the concerns, and that is why we choose nextflow, which heavily improves continuous integration. The modular code makes it very easy to include new tools or integrate tools if the users request them. Additionally, as you can directly see in the code at Github we already had some developers from popular phage prediction tools that were eager to help with implementation steps (Phigaro). Further, we went with individual containerization (docker/singularity) for each integrated bioinformatic software to avoid any typical incompatibility issues and installation problems. (containerized nextflow workflows are rather "stable" throughout the years). Therefore, we designed the workflow to be as future-proof as possible and as easy to main as possible. We also considered good coding practice and code readability aspects.<br>We aim to update WtP regularly (similar to poreCov: https://github.com/replikation/poreCov) while responding to issues (e.g. see our git issue tab) (https://github.com/replikation/What_the_Phage/issues; https://github.com/replikation/What_the_Phage/issues?q=is%3Aissue+is%3Aclosed). In the next few months, we will write a tutorial on how to integrate new tools, so developers do not rely completely on us.<br><br>Some tools (e.g., Virsorter2) can be used to predict viruses out from common bacteriophages, e.g., NCLDV and virophage (See the original article of VirSorter2). Those kinds of viruses should be described briefly in this section as well as common dsDNA phages.<br><br>Thank you for the suggestion. We updated the information in our manuscript:<br><br>"The dsDNA-tailed phages, possibly make up the majority of phages on the planet [3]. Single-stranded DNA (ssDNA), single-stranded RNA (ssRNA), and double-stranded RNA viruses are minor groups [4]."<br><br>Assembly-free long read is described here, but I think this is a bit far from the scope of this manuscript. Indeed, the dataset used in this study (ERR575692) is derived from Illumina HiSeq and the performance of assembly-free long-read dataset was not analyzed in this study. I think the descriptions could be moved to the Discussion section rather than the Introduction. Rather than that, it would be better to add more attractive descriptions about studies of phage genomes identified from short-read metagenomes to emphasize the importance of phage prediction and the value of the proposed tool, WtP. e.g., History of viral genomics using metagenomic dataset, recent technical improvement of metagenomics, phylogenetic diversity of phages, discovery of novel and uncharacterized phages from environmental metagenome, etc.<br><br>Thank you for the suggestion. We agree that it is far from the scope of our manuscript. The sentence was intended to show that with long-read sequencing technologies, especially with the MinION device, everyone can sequence phages everywhere (on site) and therefore find more phages in the future. We clarified this in the introduction

Powered by Editorial Manager® and ProduXion Manager® from Aries Systems Corporation

and data description section:

“It should be noted that due to the genome size of phages ranging from 5 kbp to 500 kbp [10], their entire genome can be sequenced via long-read technologies (e.g., Oxford Nanopore Technologies or PacBio) [11]. These sequencing techniques facilitate phage genome recovery in their natural habitat without the need to culture their hosts to isolate the phages [2] and sequencing of soil or ocean samples on-site, e. g., with the portable MinION sequencing device. Such technological developments led to a rapid increase in human gut virome studies [12] and the discovery of novel, uncharacterized phages from environmental metagenomes [13,14].”

and:

“The dataset analyzed in this study (ERR575692) is derived from Illumina HiSeq data”

Only 5 out of 11 tools that used in WtP were introduced here. The remaining 6 tools would be better to also cite here with a brief explanation of those strategies for virus prediction.

Thank you for the suggestion. We updated the information in our manuscript:

“Existing prediction tools rely on direct comparison of sequence similarity [15,16], sequence composition [17,18], and models based on these features derived through learning algorithms [15,16,19]. The phage prediction tool DeepVirFinder uses a k-mer-based deep learning method using convolutional neural networks and builds on its predecessor VirFinder [18,20]. PPR-Meta also utilizes convolutional neural networks to identify both phages and plasmids [19]. Metaphinder integrates BLAST hits to multiple genomes in a database to identify phage sequences in assembled contigs [21]. Seeker and VirNet work with a deep learning framework that uses long short-term memory models that do not depend on sequence motives [22,23], while Vibrant utilizes deep learning neural networks based on protein signatures [15]. Virsorter2 builds on the strategy of Virsorter (first iteration) by applying machine learning to evaluate the viral content using genomic features [16,24]. Phigaro uses pre-computed sets of pVOG profile HMMs (Hidden Markow Models) [25]”

Also, MARVEL was cited here but not used in WtP.

Thank you for the suggestion. We removed MARVEL-related information in our manuscript.

Figure 1 is different from the one on the GitHub page (https://mult1fractal.github.io/wtp-documentation/figures/wtp-flowchart-simple.png), which seem to be better than the Figure 1.
What 'DAG' means?

Thank you for the suggestion. We updated the figure and caption in our manuscript:

“Figure 1: Simplified “What the Phage” workflow Flowchart. Sequence input (yellow) can either be first-run through the “prediction” and subsequently “Annotation & Taxonomy” as a whole or used directly as an input for the “Annotation & Taxonomy” only. Each of the multiple phage prediction tools can be individually controlled if needed (tool control).”

To clarify: DAG stands for direct acyclic graph and is a Flowchart of the processes and tools WtP utilizes.

'a metagenome assembly' could rephrase like 'metagenomic assembled contigs'

Powered by Editorial Manager® and ProduXion Manager® from Aries Systems Corporation

	Thank you for the suggestion. We changed the information to:<br><br>"The first stage takes a multi-fasta file as input (e.g., a metagenomic assembled contigs), formats it to the demands of each tool, and filters sequences below a user-defined length threshold (1,500 bp by default) via SeqKit v0.10.1 [28]"<br><br>Metaphinder and Seeker are here with 'no release version'. I understand the situation but I feel this description is not good for reproducing the analysis. To specify the version of tools even if lack the official release version, mention the last commit date (For Metaphinder, Aug 10, 2021) or GitHub commit ID (bebc447d00ec9ff9f4960f38b627d8651262ff72) is likely a good way.<br><br>Thank you for the suggestion. The tool container that we used are already "locked in" on a specific commit id, but we completely agree that its important information for the manuscript:<br><br>"The phage prediction process is performed by eleven different tools (14 approaches) in parallel: VirFinder v1.1 [18], PPR-Meta v1.1 [19], VirSorter v1.0.6 (with and without virome mode) [16], DeepVirFinder v1.0 [20], Metaphinder with no release version (using default database and own database (Zheng et al. database, Github commit ID bebc447d00ec9ff9f4960f38b627d8651262ff72) [21], sourmash v2.0.1 [17], Vibrant v1.2.1 (with and without virome mode) [15], VirNet v0.1 [23] Phigaro v2.2.6 [25], Virsorter2 v2.0 [24] and Seeker [22] with no release version (Github commit ID 9ae14887dcd4295f4340626d06d8848cead2102d)."<br><br>In this manuscript, Prodigal was used for gene prediction. However, accurate gene prediction from phage genome is still difficult (see https://academic.oup.com/bioinformatics/article/35/22/4537/5480131). This fact have been affect both the phage prediction and functional gene annotation in the field of virology. I think the difficulty of gene prediction from phage genome and potential room for improvement should be noted in the discussion section.<br><br>Thank you for the suggestion. We added the information to our manuscript in the limitations section.<br><br>"Furthermore, WtP uses default database(s) or the original trained model(s) provided with each stand-alone prediction tool. We note that most casual users are unlikely to retrain these tools before their use.<br>Accurate gene prediction from the phage genome is still difficult [41]. This fact has affected both phage prediction and functional gene annotation in virology. New phage gene databases and algorithms could improve the quality of gene prediction in the future. We, therefore, implemented the function to provide, e.g., more recent databases to improve gene annotation."<br>We also updated the WtP-code (WtP v1.2.0) so users can provide their own gene annotation databases<br><br>The sentence ' ~ IMG/VR, iVirus, or VERVE-NET' here should be with appropriate citations or URLs. I found a paper of iVirus.<br><br>Thank you for the suggestion. We updated it in our manuscript:<br><br>"All the results are individually filterable so that the user can consider additional insights or information provided by community platforms such as IMG/VR [34] or iVirus [35]."<br><br>WTP -> WtP<br><br>Thank you for the suggestion. We updated it in our manuscript.

Powered by Editorial Manager® and ProduXion Manager® from Aries Systems Corporation

Figure 3. X-axis title of left-bottom bar plot and Y-axis title of top-right bar plot. viral -> phage

Thank you for the suggestion. We updated the figure and the WtP-code:

What 'prediction values' mean? Are these scores generated by each prediction tool?

Thank you for the suggestion. We clarified the meaning of prediction values in our manuscript:

“In general, the prediction values (p-values, scores and outputs generated by the phage prediction tools) are above >0.7 for the first four sequences/contigs (NODE_14, NODE_13, NODE_12, NODE_30), indicating high consensus among the prediction tools, although in some cases tools prediction values were below 0.5 (Phigaro: NODE_30, Seeker: NODE_12 and NODE_30, Virnet: NODE_12 and Virsorter2: NODE_30).”

Figure 4. X-axis texts. Unify the format to either NodeID:assignment (e.g., NODE_5:unknown) or assignment:NodeID (T3:NODE_14).

Thank you for the suggestion. We updated it in our manuscript:

The sequences matched with 100% identity to Salmonella enterica (Salmonella enterica strain FDAARGOS_768 chromosome, complete genome), but not to prophage sequences. ' here. Does the sentence mean that the contig NODE_5 and NODE_8 were mis-predicted as prophage by CheckV?

Thank you for the suggestion. We clarified the part in our manuscript:

“These results were manually confirmed using NCBI’s blastn (nr/nt database). The sequences matched with 100% identity to Salmonella enterica (Salmonella enterica strain FDAARGOS_768 chromosome, complete genome), but not to prophage sequences. Furthermore, NODE_8 had 1.37 times the contig length of the matched reference (from CheckV), and NODE_5 had 3.24 times the contig length of the matched reference, which may have influenced the NCBI blastn search. Salmonella enterica is known to host prophages [40]; therefore, the identified prophage sequences via CheckV are plausible results.”

Table 1. completeness -> completeness (%)

Thank you for the suggestion. We updated it in our manuscript:

Phage nameContig_idGene countCheckV
quality Completeness
[%]contig length [bp]
unknown1NODE_5107Complete100.0114,288
unknown2NODE_871High-quality100.063,147
VPE25NODE_6137High-quality99.9986,514
phiX174NODE_308Medium-quality89.355,441
T3NODE_1443High-quality93.3437,380
T7NODE_1353Complete99.4839,820
P22NODE_1267Complete100.041,715

Powered by Editorial Manager® and ProduXion Manager® from Aries Systems Corporation

Add citation in the line ' At least one multitool approach was implemented on a smaller scale by Ann C. Gregory et al. (comprising only VirFinder and VirSorter). '

Thank you for the suggestion. We updated it in our manuscript:

“At least one multitool approach was implemented on a smaller scale by Ann C. Gregory et al. (comprising only VirFinder and VirSorter) [29]”

16. Lack doi.
18. Lack doi.
19. Lack doi.

Thank you for the suggestion. We updated the missing doi in our manuscript.

Reviewer #2
In this manuscript, the authors developed an integrated workflow WtP for identification, annotation and taxonomy of phage sequences. Based on Docker and Nextflow, WtP integrates 11 phage sequence identification tools (including 14 approaches), two functional annotation and taxonomy tools (Prodigal and HMMER), and a visualizing tool (chromoMap). When using WtP, it is convenient that users do not need to install each tool and can avoid the conflict between each installation package and between operating systems. Also, the WtP tool was applied to the artificial microbiome. The threshold of each phage sequence prediction tool can be manually adjusted and outputted. Annotation and taxonomy results of phage sequences can be further visualized by CheckV and by chromeMap tool. However, there are some limitations in this manuscript. For the annotation and taxonomy stage, only the Prodigal tool was used for gene prediction, and no other gene prediction tools (especially the phage-specific tools). It is necessary for an integrated workflow to include other similar tools. WtP needs at least 4 GB of memory and 75 GB of storage, so the author should develop a web version or at least a graphical interface version of WtP for its prevalence.

Major comment:
1. Except for sequence identification, host prediction (e.g., HoPhage, PHP, and VirHost Matcher-Net) and lifestyle prediction (e.g., DeepPhage, PhagePred) of phage sequences are also important in microbial communities. However, WtP did not involve those functions.

We agree that host prediction and lifestyle prediction are important parameters to describe phages, and it is correct that WtP does not include these. WtP focuses on the reliable execution of many state-of-the-art phage prediction tools to address the major bottleneck of phage identification.
We wanted to make sure that phage identification is reproducible and installation of the several included tools is not needed, as WtP takes care of all the necessary steps to install, run, and collect the tool outputs in an easy-to-read and interactive result report. From our own experience, we know how hard it is to get several phage identification tools to run in one environment when there is no bioinformatic knowledge available to help. This is also the general feedback of other users (on conferences, GitHub, and via E-mail) we receive.
We understand the concerns, and that is why we choose nextflow, which heavily improves continuous integration. The modular code makes it very easy to include new tools or integrate tools if the users request them. Further, we went with individual containerization (docker/singularity) for each integrated bioinformatic software to avoid any typical incompatibility issues and installation problems (containerized nextflow workflows are rather “stable” throughout the years). Therefore, we designed the workflow to be as future-proof as possible and as easy to maintain as possible. We also considered good coding practice and code readability aspects. We aim to update WtP regularly (similar to poreCov: https://github.com/replikation/poreCov) while responding to issues (e.g. see our git issue tab)

(https://github.com/replikation/What_the_Phage/issues; https://github.com/replikation/What_the_Phage/issues?q=is%3Aissue+is%3Aclosed). However, good coding practice and workflow design are best when focused on one problem/topic at a time. Having dedicated workflows for distinct tasks e.g. a workflow for phage prediction, a workflow for host prediction, and a workflow for lifestyle prediction, is more convenient than managing a bloated and messy workflow that includes all phage-related tools and is hard to maintain, unlike WtP.
A good compromise could be to develop a new workflow with two - three phage prediction tools, a host prediction tool, and a lifestyle prediction tool in cooperation with international phage experts.

2. In addition to the web version or graphical interface version of WtP, the author can also consider a video demo or usage illustration. To clarify the purpose of this study, I think it would be better to add the phrase 'a web server of ...' or 'a GUI platform of ...' into the title.

This would require a lot of additional and continuous funding for computer hardware and maintenance. Something like this is only possible for small-scale tools. Therefore, nearly never done for any compute-intensive bioinformatic tool or workflow. However, there is a reason we and many others are implementing workflows in nextflow as one could execute (every) nextflow workflow, such as WtP, directly via the "Tower." (https://cloud.tower.nf/). The developer of nextflow provides a "universal" GUI/Dashboard to execute nextflow workflows. Nextflow tower offers free and paid tiers and a simplistic GUI. So it's up to each user if they, e.g., want to execute WtP via a Dashboard such as Tower. There are many tutorials for this available; it basically comes down to adding the workflow you want to use and providing the compute environment (where to run, e.g., google cloud, AWS, etc.). Web hosting servers usually have a limited lifespan until they expire. Some web tools need a lot of time to upload big datasets. On the contrary, WtP can preload all necessary databases and then execute WtP without being connected to the internet at all, which is especially useful when having bad internet connections (https://github.com/replikation/What_the_Phage/issues/167).

3. In 'Analysis' Section (Page 12), only four contigs of phage sequences can be annotated in artificial data: P22 (NODE_12), T3 (NODE_14), T7 (NODE_13) and phiX174 (NODE_30). The 'predicted_organism_name' of the remaining 102 phage contigs are 'no match found'. Can WtP improve or add more databases to annotate more contigs?

We understand your concern. We described in the data description section that the artificial metagenome consists of germ-free mice feces bacteria and phage cultures:

“Kleiner et al. [38] generated an artificial microbiome via bacteria and phage cultures in mice feces (germ-free C57BL/6 J mice) and sequenced the sample. The group added six phages: P22, T3, T7, ϕ6, M13, and ϕVPE25, and two bacteria (Listeria monocytogenes and Bacteroides thetaiotaomicron) to germ-free feces. We, therefore, expect the prediction of the six known phages and possibly other phage sequences related to both bacteria strains. Still, false-positive hits and tool disagreements are plausible results during the phage prediction process.“

WtP identified all relevant phages that were also identified by Kleiner et al. . Therefore, the remaining contigs could be of Bacteria or mouse origin.
Your suggestion to add more databases for phage annotation is very good. We updated the WtP workflow (v1.2.0) so that users can provide their own annotation databases:

“Furthermore, WtP uses default database(s) or the original trained model(s) provided with each stand-alone prediction tool. We note that most casual users are unlikely to retrain these tools before their use.
Accurate gene prediction from the phage genome is still difficult [41]. This fact has affected both phage prediction and functional gene annotation in virology. New phage

gene databases and algorithms could improve the quality of gene prediction in the future. We, therefore, implemented the function to provide, e.g., more recent databases to improve gene annotation.”

4. In 'Analysis' Section (Page 14), the author mentions 'No specialized phage assembly strategy or any cleanup step was included during the assembly step'. I think it is unreasonable, and the downstream analysis will inevitably be affected by the impurity sequences.

We understand the reviewer's concerns. However, as the organisms of this dataset were known beforehand, we were able to retrieve all phages that the group of Kleiner et al. found in their analysis and also the internal Illumina control. We, therefore, think a specialized phage assembly strategy or any cleanup step was not necessary in this special case. To get rid of potential impurity, WtP filters and analyzes only sequences above 1500 bp by default (this value is adjustable).
We agree with the reviewer that if users want to analyze unknown datasets from sequencing data, phage assembly strategies (especially for Illumina sequenced datasets) are necessary to improve results.

5. In Figure 2, it is possible to export results in the form of 'csv', 'pdf' or 'excel'. Can WtP export all the predicted phage sequences in the form of 'fasta'. The author should describe how to change or add the database during the annotation and classification phases.

Yes, it is possible. In the report, above the tables, there are buttons for exporting the data in Excel, CSV, or PDF files. For better understanding, we added the information in the figure 2 caption:

“Figure 2: Example figure of the “Phage prediction by contig table”-section of the result report summarizes the tool outputs for the analyzed sample ERR575692. The full result report can be found here: https://replikation.github.io/What_the_Phage/. All tables can be exported as Excel, PDF, or CSV files by using the buttons above the tables.”

Your suggestion to add more databases for phage annotation is very good. We updated the WtP workflow (v1.2.0) so that users can provide their own annotation databases:

“Furthermore, WtP uses default database(s) or the original trained model(s) provided with each stand-alone prediction tool. We note that most casual users are unlikely to retrain these tools before their use.
Accurate gene prediction from the phage genome is still difficult [41]. This fact has affected both phage prediction and functional gene annotation in virology. New phage gene databases and algorithms could improve the quality of gene prediction in the future. We, therefore, implemented the function to provide, e.g., more recent databases to improve gene annotation.”

Can WtP export all the predicted phage sequences in the form of 'fasta'.

We understand this request. WtP collects the outputs of the phage prediction tools solely. The problem: How to set the cut-off if a sequence is a phage or not. Potential false negative or false positive sequences could be exported. Therefore we think the user should further analyze the results provided and extract the sequences of interest by themselves using the provided tutorial in the result report:
“Extract contigs of interest
Filter the Phage prediction by contig table to your liking
Click on the CSV-Button (this will download the Phage prediction by contig table)
Open your Linux-Terminal
mkdir contigs_of_interest
cd contigs_of_interest
Copy the downloaded Phage prediction by contig table to the contig_IDs_of_interest -

folder
Copy the input_fasta to the contig_IDs_of_interest -folder
cp WtP_results/your_sample/Input_fasta/your_input_fasta.fa.gz
/foo/bar/contigs_of_interest
Get contig IDs of interest
tail -n+2 final_report.utf8.csv | tr -d "" | cut -f2 -d"," > contig_IDs_of_interest.txt
via Docker: use Seqkit to extract contigs of interest of your input fasta-file
docker run --rm -it -v $PWD:/input nanozoo/seqkit:0.13.2--cd66104
cd input
seqkit grep --pattern-file contig_IDs_of_interest.txt your_input_fasta.fa.gz >
contigs_of_interest.fa
Finally, close the docker with ctrl + d"

"We think it is still the user's task to select the most likely phage contigs, extract them, and use them for a more detailed and curated analysis, based on the phage prediction tool outputs, collected in the result report. WtP can only provide an all-encompassing starting point for phage."

Minor comment:
1. In 'Functional annotation & Taxonomy' Section (Page 8), 'Figure 3' in the sentence 'All annotations are summarized in an interactive HTML file via chromoMap (see Figure 3)' should be 'Figure 4'.

Thank you for the suggestion. We updated it in our manuscript according to your suggestion:

"All annotations are summarized in an interactive HTML file via chromoMap [33] (see Figure 4)"

2. The column of 'Computeness' in Table 1 missed the unit, and the author could add an outer border to Table 1.

Thank you for the suggestion. We updated it in our manuscript according to your suggestion:

Phage nameContig_idGene countCheckV
 qualityCompleteness
[%]contig length [bp]
unknown1NODE_5107Complete100.0114,288
unknown2NODE_871High-quality100.063,147
VPE25NODE_6137High-quality99.9986,514
phiX174NODE_308Medium-quality89.355,441
T3NODE_1443High-quality93.3437,380
T7NODE_1353Complete99.4839,820
P22NODE_1267Complete100.041,715

3. Figure 2 need to be clearer.

Thank you for the suggestion. We updated it in our manuscript according to your suggestion. We clarified what the reader sees in the figures.

"Figure 2: Example figure of the "Phage prediction by contig table"-section of the result report. The "Phage prediction by contig table"-section summarizes the tool outputs for the analyzed sample ERR575692. The full result report can be found here: https://replikation.github.io/What_the_Phage/. All tables can be exported as Excel, PDF, or CSV files by using the buttons above the tables."

Powered by Editorial Manager® and ProduXion Manager® from Aries Systems Corporation

3. Figure 3 need to be clearer.
Thank you for the suggestion. We updated the figure. We also simplified the description and how to read this plot as much as possible. How to read the diagram: For example, 53 phage contigs are found by six tools (DeepVirFinder, Metaphinder-own-DB, Metaphinder, PPRmeta, seeker, and Virfinder). Another 42 non-overlapping phage contigs are found by these tools but also virnet.
We chose the UpSet diagram as it is clearer for visualizing the intersection between more than 3 sets (phage prediction tools) in comparison to standard Venn diagrams.
If the reviewer could provide a better and simpler description of this diagram, we would be happy to include this in our manuscript.

Figure 3: UpSet plot summarizing the prediction performance of each tool for the sample ERR575692. The total amount of identified phage contigs per tool is shown in blue bars on the left. Black, vertical bars visualize the number of contigs that each tool or tool combination has uniquely identified. Each tool combination is shown below the vertical barplot as a dot matrix. How to read the diagram: For example, 53 phage contigs are found by six tools (DeepVirFinder, Metaphinder-own-DB, Metaphinder, PPRmeta, Seeker, and VirFinder). Another 42 contigs are found by these tools but also virnet.

4. Page 5. 'approach to gain' should be 'approach to gaining'.

Thank you for the suggestion. We updated it in our manuscript according to your suggestion:

“Sequencing the entire DNA of environmental samples (metagenomics) is an essential approach to gaining insights into the microbiome and functional properties.”

5. Page 13. 'In addition to' should be 'In addition to'.

Thank you for the suggestion. We updated it in our manuscript according to your suggestion:

“In addition to the phages mentioned above, two more large contigs with capsid and tail gene annotations indicate prophage(s) of Salmonella enterica (contig NODE_5 and NODE_8).”

Additional Information:	
**Question**	**Response**
Are you submitting this manuscript to a special series or article collection?	No
**Experimental design and statistics**<br><br>Full details of the experimental design and statistical methods used should be given in the Methods section, as detailed in our Minimum Standards Reporting Checklist. Information essential to interpreting the data presented should be made available in the figure legends.<br><br>Have you included all the information	Yes

requested in your manuscript?	
**Resources**<br><br>A description of all resources used, including antibodies, cell lines, animals and software tools, with enough information to allow them to be uniquely identified, should be included in the Methods section. Authors are strongly encouraged to cite Research Resource Identifiers (RRIDs) for antibodies, model organisms and tools, where possible.<br><br>Have you included the information requested as detailed in our Minimum Standards Reporting Checklist?	Yes
**Availability of data and materials**<br><br>All datasets and code on which the conclusions of the paper rely must be either included in your submission or deposited in publicly available repositories (where available and ethically appropriate), referencing such data using a unique identifier in the references and in the "Availability of Data and Materials" section of your manuscript.<br><br>Have you have met the above requirement as detailed in our Minimum Standards Reporting Checklist?	Yes

Manuscript
Click here to access/download;Manuscript;What the Phage_gigascience_revision_final.docx

What the Phage: A scalable workflow for the identification and analysis of phage sequences

Mike Marquet*[1,2,3], Martin Hölzer[4], Mathias W. Pletz[1,2,3,7], Adrian Viehweger[5], Oliwia Makarewicz[1,2,3,7], Ralf Ehricht[6,7,8], Christian Brandt[1,3,7]

[1] Institute of Infectious Diseases and Infection Control, Jena-University Hospital / Friedrich Schiller University, Jena, 07747, Germany

[2] Center of Sepsis Control and Care (CSCC), Jena, Germany

[3] Leibniz Center for Photonics in Infection Research (LPI), 07747 Jena, Germany

[4] Bioinformatics and Systems Biology, Robert Koch Institute, 13353 Berlin, Germany

[5] Medical Microbiology and Virology, University Hospital Leipzig, Leipzig, 04103, Germany

[6] Leibniz Institute of Photonic Technology (Leibniz-IPHT), Jena, Germany

[7] InfectoGnostics Research Campus, Jena, Germany

[8] Institute of Physical Chemistry, Friedrich-Schiller-University Jena, Jena, Germany

* Corresponding author

E-mail addresses for all authors

Mike.marquet@med.uni-jena.de

Hoelzerm@rki.de

Mathias.Pletz@med.uni-jena.de

Adrian.Viehweger@medizin.uni-leipzig.de

Oliwia.Makarewicz@med.uni-jena.de

Ralf.Ehricht@leibniz-ipht.de

Christian.Brandt@med.uni-jena.de

ORCID iDs:

Mike Marquet [0000-0003-4344-8289]; Martin Hölzer [0000-0001-7090-8717]; Mathias W Pletz [0000-0001-8157-2753]; Adrian Viehweger [0000-0002-8970-5204]; Oliwia Makarewicz [0000-0002-4404-1862]; Ralf Ehricht [0000-0002-6612-0043]; Christian Brandt [0000-0002-7199-3957];

Abstract

Phages are among the most abundant and diverse biological entities on earth. Phage prediction from sequence data is a crucial first step to understanding their impact on the environment. A variety of bacteriophage prediction tools have been developed over the years. They differ in algorithmic approach, results, and ease of use. We, therefore, developed "What the Phage" (WtP), an easy-to-use and parallel multitool approach for phage prediction combined with an annotation and classification downstream strategy, thus, supporting the user's decision-making process by summarizing the results of the different prediction tools in charts and tables. WtP is reproducible and scales to thousands of datasets through a workflow manager (Nextflow). WtP is freely available under a GPL-3.0 license (https://github.com/replikation/What_the_Phage).

Keywords

Phage prediction, Easy-to-use, Nextflow, Docker, Multi-tool approach, Scalable

Background

Bacteriophages (phages) are viruses that infect prokaryotes and replicate by utilizing the host's metabolism[1,2]. They are among the most abundant and diverse organisms on the planet and inhabit almost every environment[2]. The dsDNA-tailed phages possibly make up the majority of phages on the planet[3] . Single-stranded DNA (ssDNA), single-stranded RNA (ssRNA), and double-stranded RNA viruses are minor groups[4].

Phages drive and maintain bacterial diversity by perpetuating the coevolutionary interactions with their bacterial prey, facilitating horizontal gene transfer and nutrient turnover through continuous cycles of predation and coevolution[5,6]. They directly impact the microbiome, e.g., the human gut, and can influence human health[7]. At the same time, phages in aquatic habitats are responsible for the mortality of nearly 20–40% of prokaryotes every day[8]. However, despite having considerable impacts on microbial ecosystems, they remain one of the least understood members of complex communities[9].

Sequencing the entire DNA of environmental samples (metagenomics) is an essential approach to gaining insights into the microbiome and functional properties.

It should be noted that due to the genome size of phages ranging from 5 kbp to 500 kbp[10], their entire genome can be sequenced via long-read technologies (e.g., Oxford Nanopore Technologies or PacBio)[11]. These sequencing techniques facilitate phage genome recovery in their natural habitat without the need to culture their hosts to isolate the phages [2] and sequencing of soil or ocean samples on-site, e. g., with the portable MinION sequencing device. Such technological developments led to a rapid increase in human gut virome studies [12] and the discovery of novel, uncharacterized phages from environmental metagenomes[13,14].

These advances demand reliable and easy-to-use phage prediction tools and workflows that can be directly used on assembled sequencing data. However, predicting phages from metagenomes and their differentiation from prophages remains a challenge as there is no established computational gold standard[13].

Existing prediction tools rely on direct comparison of sequence similarity[15,16], sequence composition[17,18], and models based on these features derived through learning algorithms[15,16,19]. The phage prediction tool DeepVirFinder uses a k-mer-based deep learning method using convolutional neural networks and builds on its predecessor VirFinder[18,20]. PPR-Meta also utilizes convolutional neural networks to identify phages and plasmids[19]. Metaphinder integrates BLAST hits to multiple genomes in a database to identify phage sequences in assembled contigs[21]. Seeker and VirNet work with a deep learning framework that uses long short-term memory models that do not depend on sequence motives[22,23], while Vibrant utilizes deep learning neural networks based on protein signatures[15]. Virsorter2 builds on the strategy of Virsorter (first iteration) by applying machine learning to evaluate the viral content using genomic features [16,24]. Phigaro uses pre-computed sets of pVOG profile HMMs (Hidden Markow Models)[25].

The performance of each prediction method varies [26] depending on the sample type or material, the sequencing technology, and the assembly method, which makes the correct choice for any given sample difficult without having to install and test several tools.

The user can choose from many tools based on different calculation strategies, software dependencies, and databases to further complicate matters. We observed various installation issues and conflicts while working with these phage prediction tools, making a multi-tool screening approach complex and time-consuming.

To overcome these obstacles and issues, we developed “What the Phage” (WtP), a reproducible, accessible, and scalable workflow utilizing the advantages of multiple prediction tools in parallel to detect and annotate phages.

Methods

Design and Implementation

WtP was implemented in Nextflow, a portable, scalable, and parallelizable workflow manager[27]. At the time of writing, eleven different tools to predict phage sequences and other annotation and classification programs are included in WtP. WtP uses so-called containers (Docker or Singularity (Apptainer)) for an installation-free workflow execution without dependency or operating system conflicts for each of the currently over 21 programs included. All containers are pre-build, version-controlled, online available at the dockerhub website, and automatically downloaded. Additionally, all nine different databases (belonging to the corresponding tools) and datasets used by the workflow are managed automatically. The modular code structure and functionalities of Nextflow and Docker/Singularity (Apptainer) allow easy integration of other phage prediction tools and additional analysis steps in future releases of the pipeline. The workflow consists of two main phases, which are executed subsequently or, if specified, individually (Figure 1):

1. Prediction: The prediction of putative phage sequences
2. Annotation & Taxonomy: The gene annotation and taxonomic classification of phage sequences

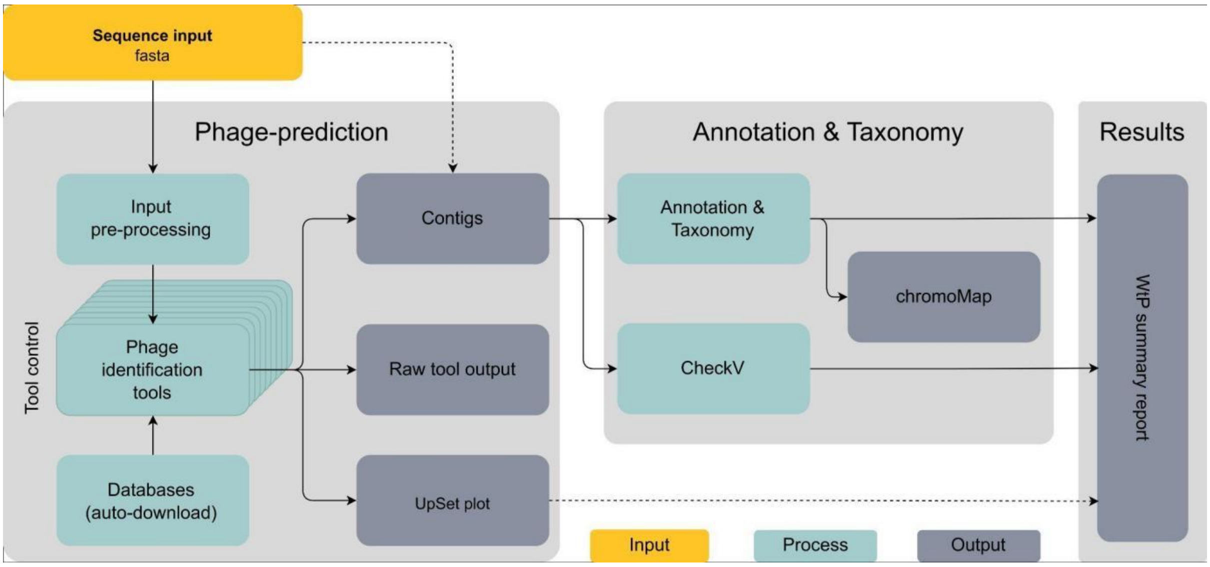

Figure 1: Simplified "What the Phage" workflow Flowchart. Sequence input (yellow) can either be first-run through the "prediction" and subsequently "Annotation & Taxonomy" as a whole or used directly as an input for the "Annotation & Taxonomy" only. Each of the multiple phage prediction tools can be individually controlled if needed (tool control).

Prediction and Visualization

The first stage takes a multi-fasta file as input (e.g., a metagenomic assembled contigs), formats it to the demands of each tool, and filters sequences below a user-defined length threshold (1,500 bp by default) via SeqKit v0.10.1 (SeqKit, RRID:SCR_018926)[28]. Sequences that are too small usually generate false-positive hits, as Gregory *et al*. [29]observed. The phage prediction process is performed by eleven different tools (14 approaches) in parallel: VirFinder v1.1[18], PPR-Meta v1.1 (PPR-Meta, RRID:SCR_016915)[19], VirSorter v1.0.6 (with and without virome mode)[16], DeepVirFinder v1.0[20], Metaphinder with no release version (using default database and own database (Zheng *et al. database*, Github commit ID bebc447d00ec9ff9f4960f38b627d8651262ff72)[21], sourmash v2.0.1[17], Vibrant v1.2.1 (with and without virome mode)[15], VirNet v0.1[23], Phigaro v2.2.6[25], Virsorter2 v2.0 [24] and Seeker [22] with no release version (Github commit ID 9ae14887dcd4295f4340626d06d8848cead2102d). Tool outputs are collected in a detailed result report (See section: Result report, Figure 2; Data availability section [30]).

Functional annotation & Taxonomy

For this step, Prodigal v2.6.3-1 (Prodigal, RRID:SCR_011936) [31]is used in metagenome mode to predict ORFs and HMMER v3.3 (Default cutoff: -E 1e-30; RRID:SCR_005305) [32]to identify homologs via the pVOG-database[33]. All annotations are summarized in an interactive HTML file via chromoMap [34] (see Figure 4). Additionally, WtP classifies all contigs

via sourmash and provides a probability score to the corresponding taxonomic classification based on Zheng *et al*. database [35].

Result report

WtP streamlines the detection of phage sequences across multiple tools in their default settings, thus balancing some drawbacks (e.g., relying on updated databases, only predicting phages available in databases). To ease the data interpretation for the user, WtP collects the results in a detailed summary report HTML file for simplified interpretation (Figure 2, Data availability section [30]). The report contains an UpSet plot summarizing the prediction performance of each tool (Figure 2). Finally, the “phage prediction by contig table” (Figure 2) summarizes tool outputs for each contig. WtP assigns numeric values to tools that do not generate p-values or scores between 0 and 1 (see result report, Phage prediction by contig section) and sorts them based on phage likelihood. All the results are individually filterable so that the user can consider additional insights or information provided by community platforms such as IMG/VR [36] or iVirus[37].

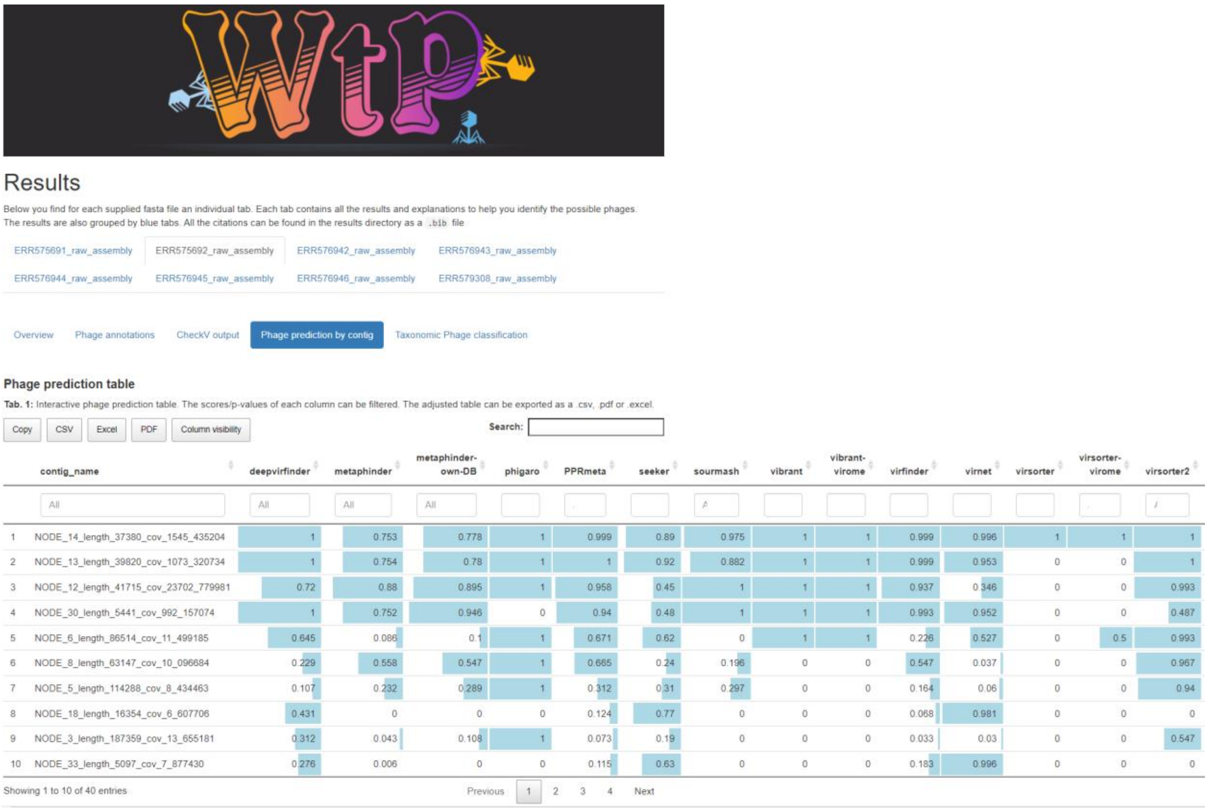

	contig_name	deepvirfinder	metaphinder	metaphinder-own-DB	phigaro	PPRmeta	seeker	sourmash	vibrant	vibrant-virome	virfinder	virnet	virsorter	virsorter-virome	virsorter2
1	NODE_14_length_37380_cov_1545_435204	1	0.753	0.778	1	0.999	0.89	0.975	1	1	0.999	0.996	1	1	1
2	NODE_13_length_39820_cov_1073_320734	1	0.754	0.78	1	1	0.92	0.882	1	1	0.999	0.953	0	0	1
3	NODE_12_length_41715_cov_23702_779981	0.72	0.88	0.895	1	0.958	0.45	1	1	1	0.937	0.346	0	0	0.993
4	NODE_30_length_5441_cov_992_157074	1	0.752	0.946	0	0.94	0.48	1	1	1	0.993	0.952	0	0	0.487
5	NODE_6_length_86514_cov_11_499185	0.645	0.086	0.1	1	0.671	0.62	0	1	1	0.226	0.527	0	0.5	0.993
6	NODE_8_length_63147_cov_10_096684	0.229	0.558	0.547	1	0.665	0.24	0.196	0	0	0.547	0.037	0	0	0.967
7	NODE_5_length_114288_cov_8_434463	0.107	0.232	0.289	1	0.312	0.31	0.297	0	0	0.164	0.06	0	0	0.94
8	NODE_18_length_16354_cov_6_607706	0.431	0	0	0	0.124	0.77	0	0	0	0.068	0.981	0	0	0
9	NODE_3_length_187359_cov_13_655181	0.312	0.043	0.108	1	0.073	0.19	0	0	0	0.033	0.03	0	0	0.547
10	NODE_33_length_5097_cov_7_877430	0.276	0.006	0	0	0.115	0.63	0	0	0	0.183	0.996	0	0	0

Figure 2: Example figure of the "Phage prediction by contig table"-section of the result report. The "Phage prediction by contig table"-section summarizes the tool outputs for the analyzed sample ERR575692. The full result report can be found in the data availability section [30]. *All tables can be exported as Excel, PDF, or CSV files by using the buttons above the tables.*

Other features

All mandatory databases and containers are automatically downloaded when the workflow is started and stored for the following executions. Additionally, the workflow can be pre-setup to analyze sequences offline subsequently. WtP provides the raw output of each tool to support a transparent and reproducible mode of operation. Maximum execution stability is ensured by automatically excluding phage prediction tools that cannot analyze the input data without failing the workflow (e.g., file too large, not the scope of an individual tool). We also provide a detailed user manual that is regularly updated [38].

Dependencies and version control

WtP requires the workflow management software Nextflow [27] and either Docker [39] or Singularity (Apptainer) [40]installed and configured on the system. The pipeline was tested on Ubuntu 16.04 LTS, Ubuntu 18.04 LTS, and Windows 10 (via Windows Subsystem for Linux 2 using Docker). The installation process is described in detail in the WtP user manual [38]. .
Each workflow release specifies the Nextflow version to avoid any version conflicts between the workflow code and the workflow manager. A specific Nextflow version can be directly downloaded as an executable file from the Nextflow website.
Additionally, each container used in the workflow is tagged by the accompanying tool version, pre-build, and stored on hub.docker.com.

Data Description

To demonstrate the utility and performance of WtP, we analyzed a described metagenome data set (ENA Study PRJEB6941, ERR575692) using a local desktop machine (24 threads, 60 GB RAM, Ubuntu 18.04.4 LTS) and WtP release v1.1.0. Kleiner *et al*. [41]generated an artificial microbiome via bacteria and phage cultures in mice feces (germ-free C57BL/6 J mice) and sequenced the sample. The group added six phages: P22, T3, T7, ϕ6, M13, and ϕVPE25, and two bacteria (*Listeria monocytogenes and Bacteroides thetaiotaomicron*) to germ-free feces. We, therefore, expect the prediction of the six known phages and possibly other phage sequences related to both bacteria strains. Still, false-positive hits and tool disagreements are plausible results during the phage prediction process. The dataset analyzed in this study (ERR575692) is derived from Illumina HiSeq data.

Analysis

The raw read data sets composed of eight samples were downloaded from the ENA server and individually assembled via metaSPAdes v3.14 using the default settings[42]. The resulting assembly files are stored in the *GigaScience* GigaDB database [43]) and were analyzed with WtP (release v1.1.0, default settings). As WtP uses multiple tools for phage prediction, an UpSet plot summarizes for each sample the performance of all approaches executed successfully (Figure 3 for sample ERR575692).

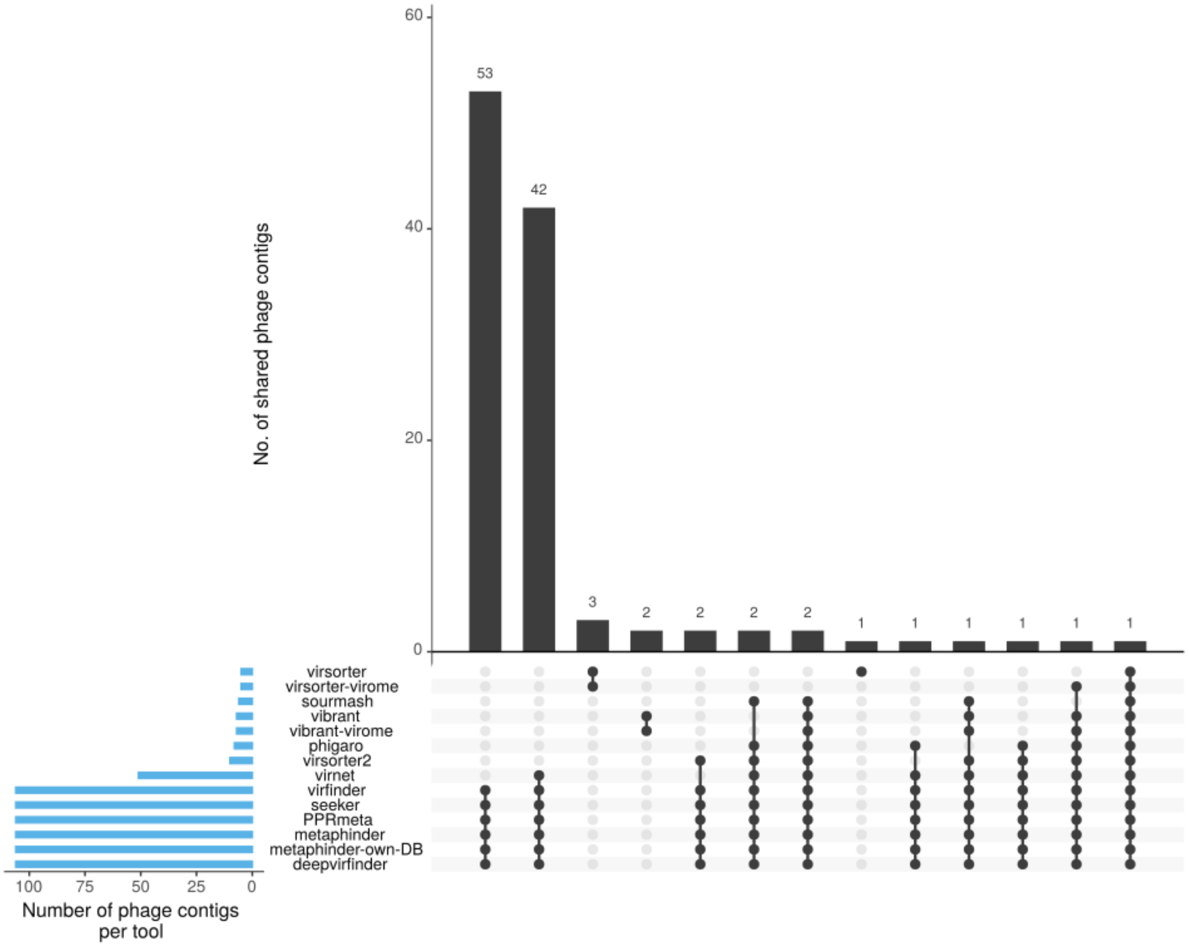

Figure 3: UpSet plot summarizing the prediction performance of each tool for the sample ERR575692. The total amount of identified phage contigs per tool is shown in blue bars on the left. Black, vertical bars visualize the number of contigs that each tool or tool combination has uniquely identified. Each tool combination is shown below the vertical barplot as a dot matrix. How to read the diagram: For example, 53 phage contigs are found by six tools (DeepVirFinder, Metaphinder-own-DB, Metaphinder, PPRmeta, Seeker, and VirFinder). Another 42 contigs are found by these tools but also Virnet.

The complete result report for sample ERR575692 can be found in the data availability section [30].

In general, the prediction values (p-values, scores and outputs generated by the phage prediction tools) are above >0.7 for the first four sequences/contigs (NODE_14, NODE_13,

NODE_12, NODE_30), indicating high consensus among the prediction tools, although in some cases tools prediction values were below 0.5 (Phigaro: NODE_30, Seeker: NODE_12 and NODE_30, Virnet: NODE_12 and Virsorter2: NODE_30). Prediction values for NODE_6 are below 0.67, and Virsorter2 and Phigaro show high values >0.99. The same applies to NODE_8 and NODE_5, indicating dissonance for these three contigs. Surprisingly, Virsorter and Virsorter-virome only predict the sequence: NODE_14 as a phage. In case of dissonance and when tools coincide, validation of contigs via phage annotations and CheckV [44] simplifies further assessment. In the case of sample ERR575692, phage genes (like tail and capsid genes) were annotated on all seven contigs
(Figure 4).

The workflow was able to detect contigs that correspond to the phages P22 (NODE_12), T3 (NODE_14), and T7 (NODE_13). In addition, the phage for the internal Illumina control (phiX174: NODE_30) was also predicted. The M13 phage [41] could not be identified as it was not assembled via metaSPAdes due to the low read-abundance and low coverages (below 0.55x, determined by Kleiner *et al.*). The same applies to phage ϕ6, which was not detectable by Kleiner *et al.* [38]. However, VPE25 (NODE_6) was initially not taxonomically classified by WtP as it was not represented in the taxonomic database (Zheng *et al.* database) at this time; however, the corresponding contig was annotated with essential phage genes (Figure 4). Therefore, the unclassified contig was analyzed manually via blastn (nr/nt database) and resulted in the genome sequence of VPE25 (PRJEB13004).

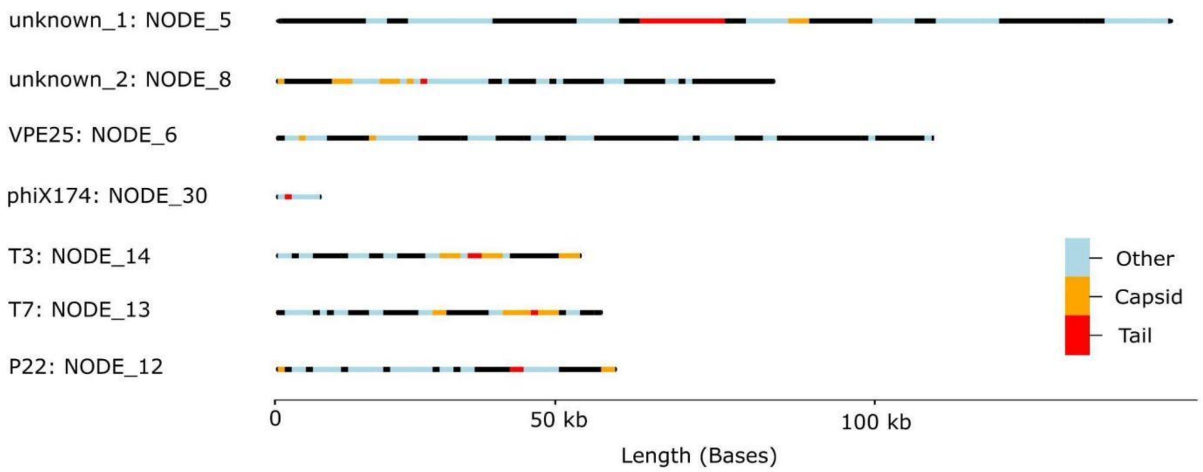

Figure 4: Visual annotation of phage contigs and annotated protein-coding genes via chromoMap. Annotations are colored based on the categories of capsid genes (orange), tail genes (red), and other genes (blue). Other contigs without either capsid or tail genes have been removed from this figure for better readability.

Furthermore, CheckV determined a phage completeness score of over 89% for all seven contigs (Table 1). In addition to the phages mentioned above, two more large contigs with capsid and tail gene annotations indicate prophage(s) of *Salmonella enterica* (contig NODE_5 and NODE_8). Both contigs showed tail and capsid genes and were labeled as prophages via CheckV with estimated completeness of over 99.99 %.

These results were manually confirmed using NCBI's blastn (nr/nt database). The sequences matched with 100% identity to *Salmonella enterica (Salmonella enterica strain FDAARGOS_768 chromosome, complete genome)*, but not to prophage sequences. Furthermore, NODE_8 had 1.37 times the contig length of the matched reference (from CheckV), and NODE_5 had 3.24 times the contig length of the matched reference, which may have influenced the NCBI blastn search. *Salmonella enterica* is known to host prophages[45]; therefore, the identified prophage sequences of CheckV are plausible results.

Table 1: Summary of the CheckV output for the sample ERR575692. All contigs with a completeness > 89 % and a length > 5,000 bp are shown.

Phage name	Contig_id	Gene count	CheckV quality	Completeness [%]	contig length [bp]
unknown1	NODE_5	107	Complete	100.0	114,288
unknown2	NODE_8	71	High-quality	100.0	63,147
VPE25	NODE_6	137	High-quality	99.99	86,514
phiX174	NODE_30	8	Medium-quality	89.35	5,441
T3	NODE_14	43	High-quality	93.34	37,380
T7	NODE_13	53	Complete	99.48	39,820
P22	NODE_12	67	Complete	100.0	41,715

Performance assessment

The WtP meta-workflow utilizes several phage identification tools and allows simple execution of a single tool or multiple tools at once. WtP does not favor or disadvantage any prediction tools based on their performance but collects each raw tool output in a user-friendly, easy-to-read result overview.

We did not additionally benchmark the tools integrated into our workflow because the performance of most of them has recently been benchmarked independently[26].

Ho *et al*. tested Virfinder v1.0, MetaPhinder, PPR_Meta v1.1, Seeker v1.0.3, Virfinder v1.1, VirSorter v1.06, VirSorter2 v2.2.3 and utilized near identical commands, parameters and databases across the benchmarked prediction tools as WtP. Another benchmark would thus only duplicate prior work.

Most tools performed well in the benchmarking of Ho *et al*., detecting the majority of phage sequences while keeping false positives low. PPR-Meta and VirSorter2, which use two different machine learning methods, had the best performance across the tools.

However, k-mer tools outperformed reference similarity and gene-based tools when tested on positive and negative phage datasets. Tests with randomly shuffled phage sequences showed a clear difference between machine learning and conventional tools.

The performance of most tools dropped significantly when a real metagenomic dataset was used compared to the RefSeq benchmark. The k-mer prediction tools showed a smaller drop in F1-score based on their RefSeq benchmark compared to reference similarity/gene-based tools as described by Ho *et al*.[26].

The group also pointed out that the tools with machine/deep learning algorithms can detect novel phages. However, their performance may be unpredictable when exposed to novel data with features that differ from those used in the training sets[26].

Therefore, we believe that a combination of phage prediction algorithms (machine/deep learning and similarity/gene-based) is a good compromise for unknown and novel datasets.

WtP deploys the benchmarked tools by default (WtP v1.2.0). If users wish to deploy other tools that were not benchmarked by Ho *et al*.[26], they can activate them easily.

While a sensical approach, multiple tools can be combined in the prediction step to yield an “ensemble” approach. To benchmark this approach, however, against individual tools is beyond the scope of this work, which aimed to facilitate the accessibility to phage prediction tools.

Limitations

Some limitations must be noted. No specialized phage assembly strategy or any cleanup step was included during the assembly step. Therefore, some smaller mice host contigs (below 5,000 bp) produced false positive hits. However, these contigs were distinguishable after the "Annotation & Taxonomy" step both in CheckV and due to the lack of typical genes related to, e.g., capsid or tail proteins, showing the application of WtP also for contaminated datasets. WtP does not filter the output of phage prediction tools for prophages, although the CheckV output indicates if a contig could be a prophage.

Furthermore, WtP uses default database(s) or the original trained model(s) provided with each stand-alone prediction tool. We note that most casual users are unlikely to retrain these tools before their use.

Accurate gene prediction from the phage genome is still difficult[46]. This fact has affected both phage prediction and functional gene annotation in virology. New phage gene databases and algorithms could improve the quality of gene prediction in the future. We, therefore, implemented the function to provide, e.g., more recent databases to improve gene annotation.

Discussion & potential implications

With the rise of metagenomics and the application of machine learning principles for virus detection, several phage prediction tools have been released over the last few years. All these tools utilize a variety of prediction approaches, all with advantages and limitations[26]. The user's choice for using certain tools often depends strongly on their usability and accessibility and less on performance. While some tools already come with a packaging system such as Conda or a containerized environment, there exists no general framework for their execution database dependencies, and installation issues prevent many potential

users from using certain tools. At least one multitool approach was implemented on a smaller scale by Ann C. Gregory *et al*. (comprising only VirFinder and VirSorter)[29]. The overarching goal of WtP is to make phage prediction tools more accessible for a broader user spectrum and non-bioinformaticians, as culture-free sequencing has led to the rapid increase of phage studies[12]. WtP acts as an ideal, all-encompassing starting point for any given assembly and provides a searchable and filterable report of the analyzed data. The user is provided with sufficient processed data (such as tool performance comparisons, taxonomic assessments, and annotation maps) to work reliably with the predicted sequences and support the decision-making process if different prediction tools are not in agreement with each other.

The meta-tool WtP allows the user to deploy current state-of-the-art phage prediction tools very easily, all at once, or only a selection of tools. WtP does not favor or disadvantage any prediction tools based on their performance analyzed in the benchmarking work of Ho *et al*.[26]. It is still the user's task to select the most likely phage contigs, extract them from WtPs output, and use them for a more detailed and curated analysis.

Further information and guides are provided either via the report or the hosted manual. WtP streamlines the prediction of phage sequence recognition across multiple tools in a reproducible and scalable workflow to allow researchers to focus on their scientific questions instead of software implementations.

Future directions

WtP is a workflow project that will be improved and extended as the modular approach and containerization simplify the integration of new tools. The predictive scope of WtP can be extended to other viruses (such as RNA viruses) and prophages by including future tools specifically designed for such use cases and adjusting filter and annotation steps. The modular nature of the workflow using Docker and Nextflow allows the integration of new phage

prediction tools by request of users, thus allowing WtP to keep up with the fast-developing field of bioinformatic tools. The versioning of WtP represents a well-functioning approach with tested and up-to-date versions of the workflow. Thus, the correct functioning of the workflow is always guaranteed and allows a reliable and fast prediction of phage sequences.

Declarations

Availability of supporting source code and requirements

Project name: What the Phage (WtP)

Project homepage: https://github.com/replikation/What_the_Phage

Programming language: Nextflow, Bash, Python, R

Other requirements: Ubuntu 18.04 LTS, Docker-version 20.10.12, Nextflow-version 21.10.6

License: GPL-3.0

RRID: SCR_022871

Data Availability

The WtP user manual [38].and Result Report are available in GitHub [30]. The WtP result data storage [47] and WtP databases are available in OSF [48]. Data used in this work is available in GitHub [49]. All supporting data and materials are available in the *GigaScience* GigaDB database [43].

List of abbreviations

WtP	What the Phage

Competing interests

None to declare.

Authors' contributions

Conceptualization, design, implementation, and Experiment conduction by M.M. and C.B. Figures created by M.M, C.B. All authors actively participated in the writing and editing of the manuscript. All authors have read and agreed to the published version of the manuscript.

Funding

This project was funded by the Federal Ministry of Education and Research (BMBF, Germany) in the framework of the Integrated Research and Treatment Centres program via the Center for Sepsis Control and Care (CSCC), Grand No. 01EO1502 (PI: MWP). MM was funded within this project. CB and RE were funded by a collaborative R&D project (BMBF) Grand No. 3GW0423B (PI: OM) and 3GW0423C (PI: RE). Funding for open-access charge: The Open Access Publishing program of the German Research Foundation (DFG) via the Thuringian University and State Library (ThULB).

.

Acknowledgments

We thank Michael Shamash for his help in properly testing and validating WtP on a Slurm-based HPC utilizing Singularity(Apptainer), Luiz Irber, to improve the sourmash integration. We also thank Polina Tikhonova and Nikos P. for their help in implementing their phage prediction tools Phigaro and Seeker.

References

1. Tulio Pardini G M, Silva B L, Aguiar A LA, Elisa Soto L M. Bacteriophage Genome Sequencing: A New Alternative to Understand Biochemical Interactions between Prokaryotic Cells and Phages. *J Microb Biochem Technol*. 2017; doi: 10.4172/1948-5948.1000362.
2. Clokie MR, Millard AD, Letarov AV, Heaphy S. Phages in nature. *Bacteriophage*. 2011; doi: 10.4161/bact.1.1.14942.
3. Fokine A, Rossmann MG. Molecular architecture of tailed double-stranded DNA phages. *Bacteriophage*. 2014; doi: 10.4161/bact.28281.
4. Ackermann H-W. Phage Classification and Characterization. In: Clokie MRJ, Kropinski AM, editors. *Bacteriophages Methods Protoc Vol 1 Isol Charact Interact*. Totowa, NJ: Humana Press; doi:10.1007/978-1-60327-164-6_13.
5. Reyes A, Semenkovich NP, Whiteson K, Rohwer F, Gordon JI. Going viral: next-generation sequencing applied to phage populations in the human gut. *Nat Rev Microbiol*. 2012; doi: 10.1038/nrmicro2853.
6. De Sordi L, Lourenço M, Debarbieux L. The Battle Within: Interactions of Bacteriophages and Bacteria in the Gastrointestinal Tract. *Cell Host Microbe*. 2019; doi: 10.1016/j.chom.2019.01.018.
7. Divya Ganeshan S, Hosseinidoust Z. Phage Therapy with a Focus on the Human Microbiota. *Antibiotics*. 2019; doi: 10.3390/antibiotics8030131.
8. Suttle CA. Marine viruses--major players in the global ecosystem. *Nat Rev Microbiol*. 2007; doi: 10.1038/nrmicro1750.
9. Sutton TDS, Hill C. Gut Bacteriophage: Current Understanding and Challenges. *Front Endocrinol*. 102019; doi: 10.1038/nrmicro1750.
10. Hatfull GF, Hendrix RW. Bacteriophages and their genomes. *Curr Opin Virol*. 2011; doi: 10.1016/j.coviro.2011.06.009.
11. Beaulaurier J, Luo E, Eppley JM, Uyl PD, Dai X, Burger A, et al.. Assembly-free single-molecule sequencing recovers complete virus genomes from natural microbial communities. *Genome Res*. 2020; doi: 10.1101/gr.251686.119.
12. Garmaeva S, Sinha T, Kurilshikov A, Fu J, Wijmenga C, Zhernakova A. Studying the gut virome in the metagenomic era: challenges and perspectives. *BMC Biol*. 2019; doi: 10.1186/s12915-019-0704-y.
13. Overholt WA, Hölzer M, Geesink P, Diezel C, Marz M, Küsel K. Inclusion of Oxford Nanopore long reads improves all microbial and viral metagenome-assembled genomes from a complex aquifer system. *Environ Microbiol*. 2020; doi: 10.1111/1462-2920.15186.
14. Ciuffreda L, Rodríguez-Pérez H, Flores C. Nanopore sequencing and its application to the study of microbial communities. *Comput Struct Biotechnol J*. 2021; doi: 10.1016/j.csbj.2021.02.020.
15. Kieft K, Zhou Z, Anantharaman K. VIBRANT: automated recovery, annotation and curation of microbial viruses, and evaluation of viral community function from genomic sequences. *Microbiome*. 2020; doi: 10.1186/s40168-020-00867-0.
16. Roux S, Enault F, Hurwitz BL, Sullivan MB. VirSorter: mining viral signal from microbial genomic data. *PeerJ*. 2015; doi: 10.7717/peerj.985.
17. Brown CT, Irber L. sourmash: a library for MinHash sketching of DNA. *J Open Source Softw*. 2016; doi: 10.21105/joss.00027.

18. Ren J, Ahlgren NA, Lu YY, Fuhrman JA, Sun F. VirFinder: a novel k-mer based tool for identifying viral sequences from assembled metagenomic data. *Microbiome*. 2017; doi: 10.1186/s40168-017-0283-5.
19. Fang Z, Tan J, Wu S, Li M, Xu C, Xie Z, et al.. PPR-Meta: a tool for identifying phages and plasmids from metagenomic fragments using deep learning. *GigaScience*. 2019; doi: 10.1093/gigascience/giz066.
20. Ren J, Song K, Deng C, Ahlgren NA, Fuhrman JA, Li Y, et al.. Identifying viruses from metagenomic data using deep learning. *Quant Biol*. 2020; doi: 10.1007/s40484-019-0187-4.
21. Jurtz VI, Villarroel J, Lund O, Larsen MV, Nielsen M. MetaPhinder—Identifying Bacteriophage Sequences in Metagenomic Data Sets. *PLOS ONE*. 2016; doi: 10.1371/journal.pone.0163111.
22. Auslander N, Gussow AB, Benler S, Wolf YI, Koonin EV. Seeker: alignment-free identification of bacteriophage genomes by deep learning. *Nucleic Acids Res*. 2020; doi: 10.1093/nar/gkaa856.
23. Abdelkareem AO, Khalil M, Elaraby M, Abbas HM, Elbehery AH. VirNet: Deep attention model for viral reads identification. *2018 13th Int Conf Comput Eng Syst ICCES*. 2018; doi: 10.1109/ICCES.2018.8639400.
24. Guo J, Bolduc B, Zayed AA, Varsani A, Dominguez-Huerta G, Delmont TO, et al.. VirSorter2: a multi-classifier, expert-guided approach to detect diverse DNA and RNA viruses. *Microbiome*. 2021; doi: 10.1186/s40168-020-00990-y.
25. Starikova EV, Tikhonova PO, Prianichnikov NA, Rands CM, Zdobnov EM, Ilina EN, et al.. Phigaro: high-throughput prophage sequence annotation. *Bioinforma Oxf Engl*. 2020; doi: 10.1093/bioinformatics/btaa250.
26. Ho SFS, Wheeler N, Millard AD, Schaik W van. Gauge your phage: Benchmarking of bacteriophage identification tools in metagenomic sequencing data. bioRxiv 2022; doi: 10.1101/2021.04.12.438782.
27. Di Tommaso P, Chatzou M, Floden EW, Barja PP, Palumbo E, Notredame C. Nextflow enables reproducible computational workflows. *Nat Biotechnol*. 2017; doi: 10.1038/nbt.3820.
28. Shen W, Le S, Li Y, Hu F. SeqKit: A Cross-Platform and Ultrafast Toolkit for FASTA/Q File Manipulation. *PLOS ONE*. Public Library of Science; 2016; doi: 10.1371/journal.pone.0163962.
29. Gregory AC, Zayed AA, Conceição-Neto N, Temperton B, Bolduc B, Alberti A, et al.. Marine DNA Viral Macro- and Microdiversity from Pole to Pole. *Cell*. 2019; doi: 10.1016/j.cell.2019.03.040.
30. Marquet M. final_report.utf8. https://replikation.github.io/What_the_Phage/. Accessed 2022 Oct 11.
31. Hyatt D, Chen G-L, LoCascio PF, Land ML, Larimer FW, Hauser LJ. Prodigal: prokaryotic gene recognition and translation initiation site identification. *BMC Bioinformatics*. 2010; doi: 10.1186/1471-2105-11-119.
32. Wheeler TJ, Eddy SR. nhmmer: DNA homology search with profile HMMs. *Bioinformatics*. 2013; doi: 10.1093/bioinformatics/btt403.
33. Grazziotin AL, Koonin EV, Kristensen DM. Prokaryotic Virus Orthologous Groups (pVOGs): a resource for comparative genomics and protein family annotation. *Nucleic Acids Res*. 2017; doi: 10.1093/nar/gkw975.
34. Anand L, Rodriguez Lopez CM. ChromoMap: an R package for interactive visualization of multi-omics data and annotation of chromosomes. *BMC Bioinformatics*. 2022; doi: 10.1186/s12859-021-04556-z.

35. Zheng T, Li J, Ni Y, Kang K, Misiakou M-A, Imamovic L, et al.. Mining, analyzing, and integrating viral signals from metagenomic data. *Microbiome*. 2019; doi: 10.1186/s40168-019-0657-y.

36. Roux S, Páez-Espino D, Chen I-MA, Palaniappan K, Ratner A, Chu K, et al.. IMG/VR v3: an integrated ecological and evolutionary framework for interrogating genomes of uncultivated viruses. *Nucleic Acids Res*. 2021; doi: 10.1093/nar/gkaa946.

37. Bolduc B, Zablocki O, Guo J, Zayed AA, Vik D, Dehal P, et al.. iVirus 2.0: Cyberinfrastructure-supported tools and data to power DNA virus ecology. *ISME Commun*. 2021; doi: 10.1038/s43705-021-00083-3.

38. Marquet M: What the Phage, User-manual. https://mult1fractal.github.io/wtp-documentation/ (2022). Accessed 2022 Oct 15.

39. Boettiger C. An introduction to Docker for reproducible research. *ACM SIGOPS Oper Syst Rev*. 2015; doi: 10.1145/2723872.2723882.

40. Kurtzer GM, Sochat V, Bauer MW. Singularity: Scientific containers for mobility of compute. *PLOS ONE*. 2017; doi: 10.1371/journal.pone.0177459.

41. Kleiner M, Hooper LV, Duerkop BA. Evaluation of methods to purify virus-like particles for metagenomic sequencing of intestinal viromes. *BMC Genomics*. 2015; doi: 10.1186/s12864-014-1207-4.

42. Nurk S, Meleshko D, Korobeynikov A, Pevzner PA. metaSPAdes: a new versatile metagenomic assembler. *Genome Res*. 2017; doi: 10.1101/gr.213959.116.

43. Mike M, Hölzer Martin, Pletz W Mathias, Viehweger Adrian, Makarewicz Oliwia, Ehricht Ralf, et al.. Supporting data for “What the Phage: A scalable workflow for the identification and analysis of phage sequences.” GigaScience Database 2022 http://doi.org/10.5524/102325

44. Nayfach S, Camargo AP, Schulz F, Eloe-Fadrosh E, Roux S, Kyrpides NC. CheckV assesses the quality and completeness of metagenome-assembled viral genomes. *Nat Biotechnol*. 2021; doi: 10.1038/s41587-020-00774-7.

45. Wahl A, Battesti A, Ansaldi M. Prophages in Salmonella enterica: a driving force in reshaping the genome and physiology of their bacterial host? *Mol Microbiol*. 2019; doi: 10.1111/mmi.14167.

46. McNair K, Zhou C, Dinsdale EA, Souza B, Edwards RA. PHANOTATE: a novel approach to gene identification in phage genomes. *Bioinformatics*. 2019; doi: 10.1093/bioinformatics/btz265.

47. Marquet M. (2022, March 2). What the Phage test profile results. https://osf.io/kuc96

48. Marquet M. (2020, November 9). What the Phage Database Storage. https://doi.org/10.17605/OSF.IO/WTFRC

49. Marquet M. Sequence data. https://github.com/mult1fractal/WtP_test-data. Accessed 2022 Oct 11.

Figure 1: Simplified "What the Phage" workflow Flowchart. Sequence input (yellow) can either be first-run through the "prediction" and subsequently "Annotation & Taxonomy"

Click here to access/download;Figure;horizontal-map-Page-1_figure1_august.jpg

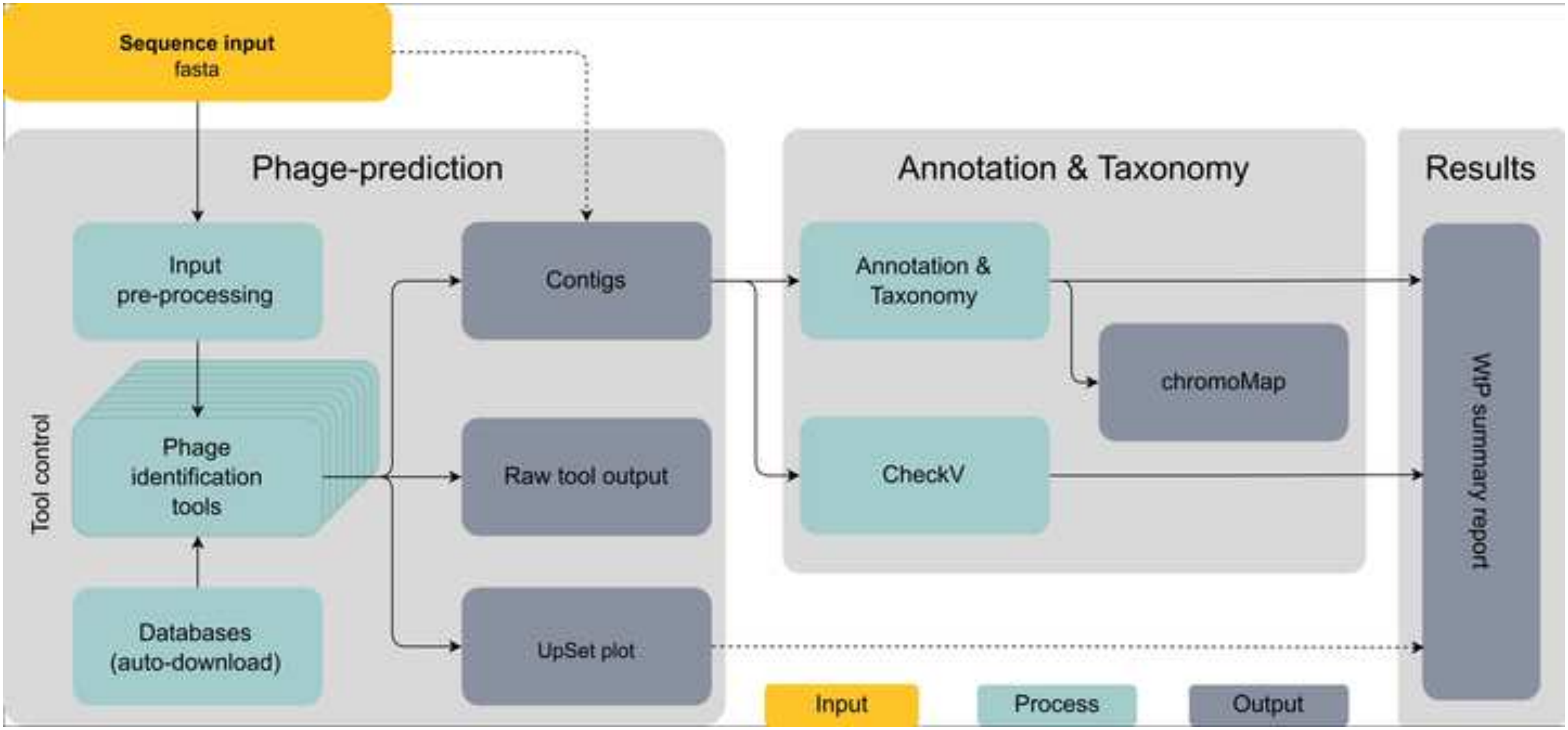

Figure 2: Example figure of the "Phage prediction by contig table"-section of the result report. The "Phage prediction by contig table"-section summarizes the tool outputs for

Click here to access/download;Figure;Result_report_example_figure_2.PNG

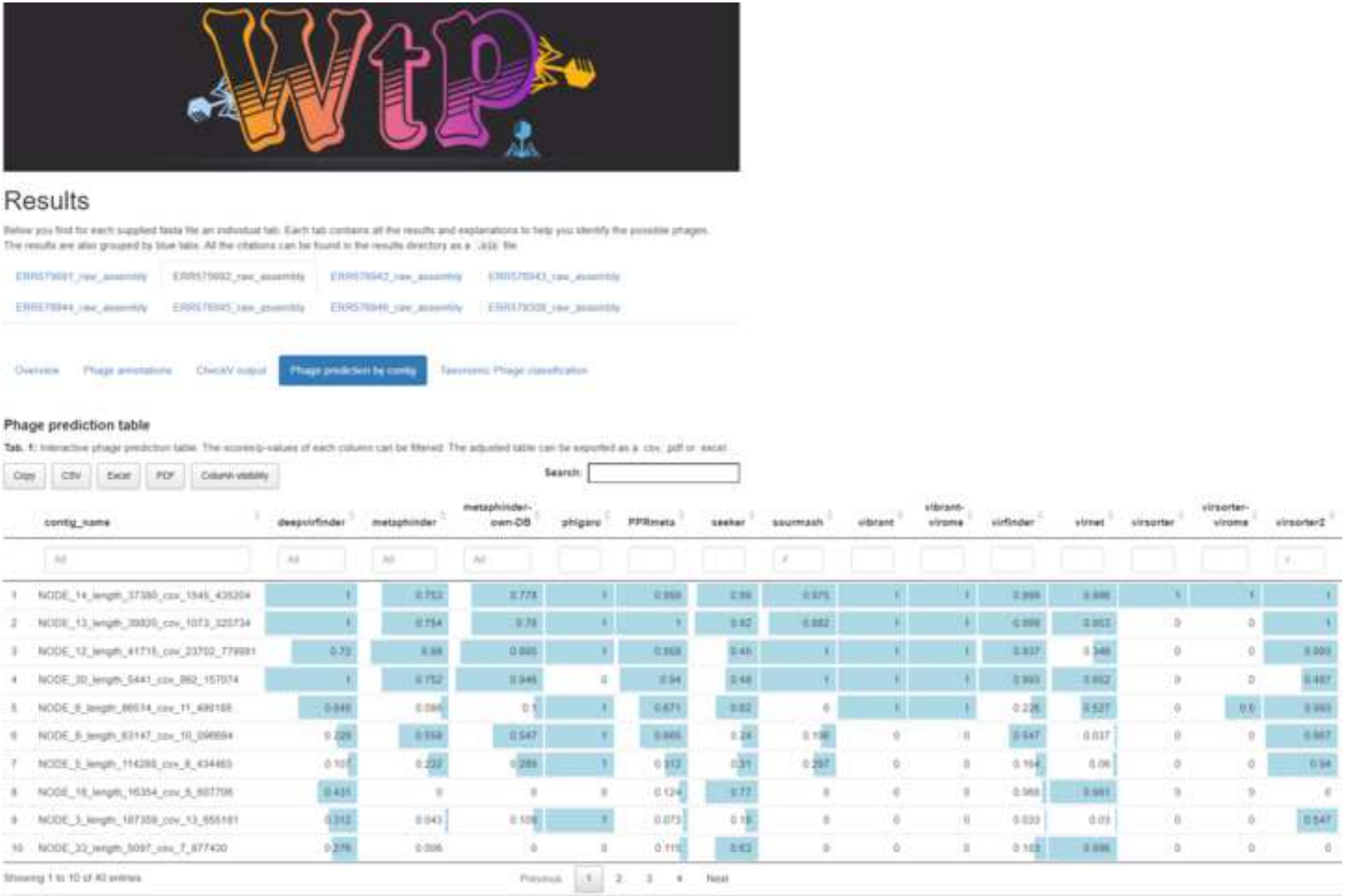

Figure 3: UpSet plot summarizing the prediction performance of each tool for the sample ERR575692. The total amount of identified phage contigs per tool is shown in

Click here to access/download;Figure;upsetr_600dpi.jpg

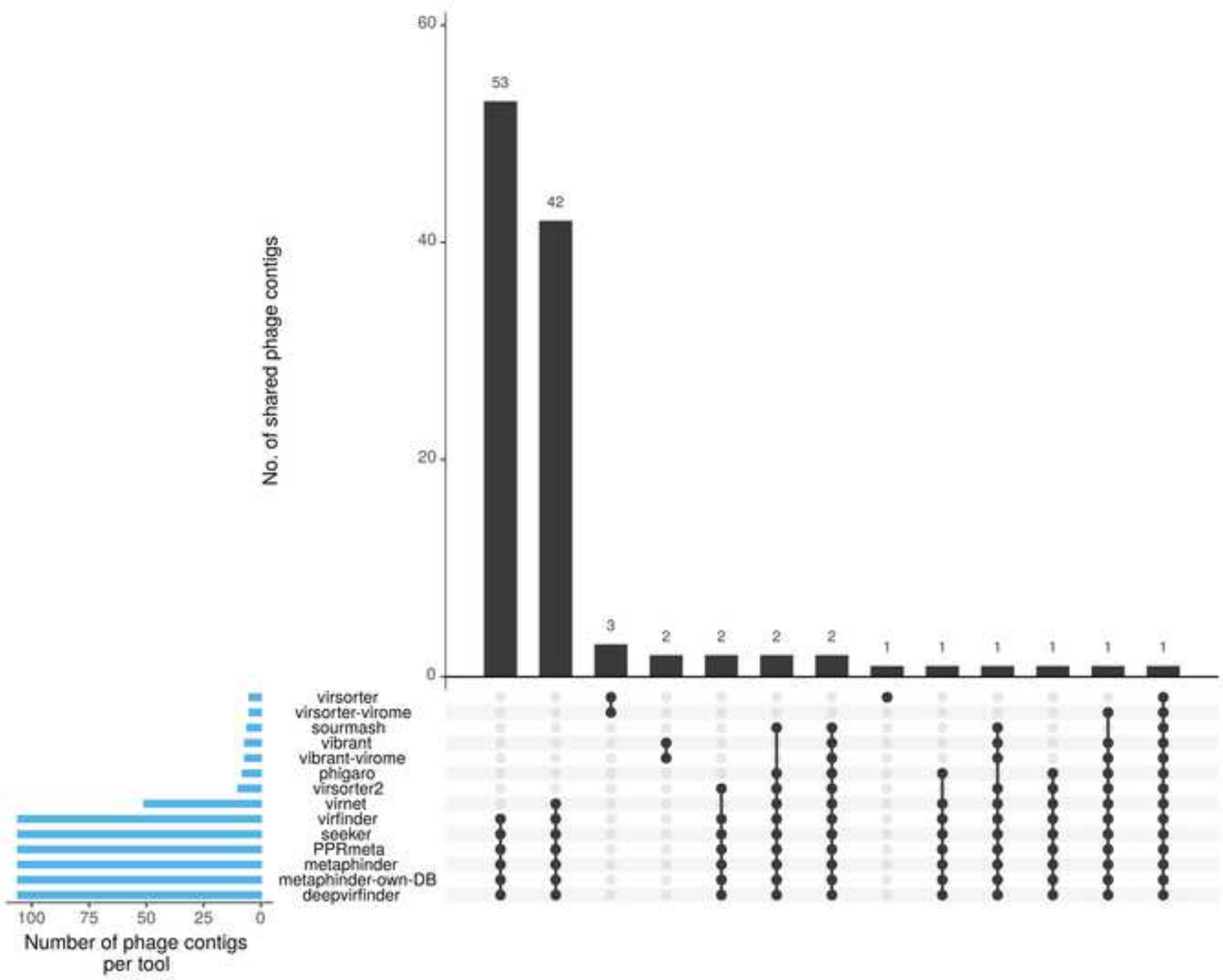

Figure 4: Visual annotation of phage contigs and annotated protein-coding genes via chromoMap. Annotations are colored based on the categories of capsid genes

Click here to access/download;Figure;WtP-figure4clean.jpg

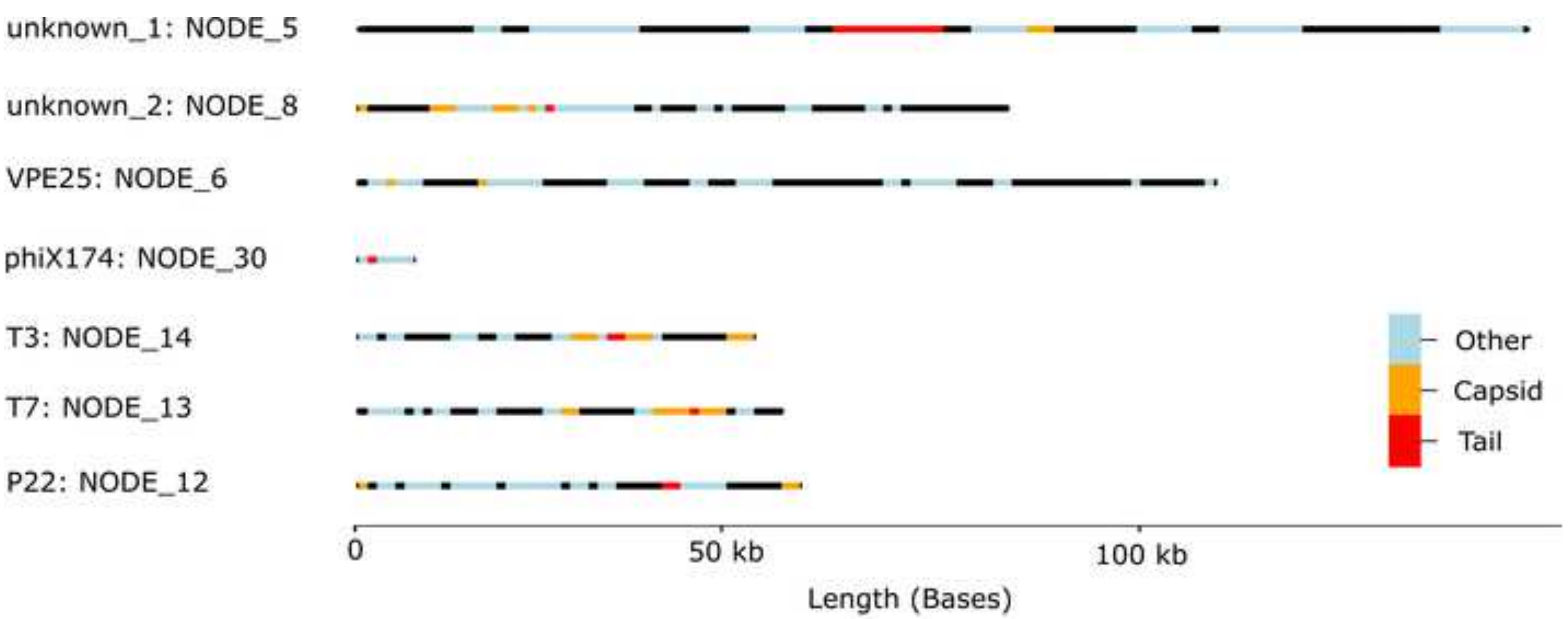

Revision requested by Reviewers

Click here to access/download
Supplementary Material
Revision for wtp.pdf
