# Supplementary material for: What the Phage: a scalable workflow for the identification and analysis of phage sequences
Source: Gigascience. 2022 Nov 18;11:giac110. doi: 10.1093/gigascience/giac110 (PMC9673492; doi:10.1093/gigascience/giac110)
Supplement: giac110_Response_to_Reviewer_Comments_Original_Submission [file giac110_response_to_reviewer_comments_original_submission.pdf]

Author's Response To Reviewer Comments

Close

Revision for “What the Phage: A scalable workflow for the identification and analysis of phage sequences”

Dear Editor and Reviewers,

First and foremost, we thank the reviewers and the editor for taking the time to look through our manuscript. The suggestions were excellent and meaningful. We addressed all the comments, and below you will find our point-by-point response to the reviewer’s comments. We feel the manuscript has improved because of these changes.

Reviewer #1
In this manuscript, the authors developed a new tool, What the Phage (WtP), for comparison of the output from multiple bioinformatics tools to predict phage sequences from genomic or metagenomic datasets. The purpose of this study is some or less meaningful. As the authors described in the Introduction section, currently it is difficult to predict reliable viral genomes, especially from culture-independent metagenomic datasets precisely because of the lack of knowledge about viral genomes in current protein/genome databases. There are many bioinformatics tools already proposed and some of them are widely used in microbiology, however, the outputs from these tools are frequently varied and conflicted among them. However, there is no good integrative platform to compare the outputs. Here, the proposed tool easily generates well-summarized output derived from multiple tools, and thus, the tool might be facilitated the analysis of phage prediction in the field of microbiology. Indeed, the authors conducted (only but) one case study using real phage genomes and reported reasonable performance. I feel the tool has some potential to contribute to the wide fields of viral genomics.

However, the user of this tool should keep in mind the fact that the tool just summarizes the output of multiple phage-prediction tools, meaning does not evaluate the reliability of the output, as described in the Discussion section. I feel thus the tool sometimes may lead to misunderstandings or make the users confuse rather than help them. It should emphasize that the majority decision among the multiple tools does not always bring the best result. The users may need further detailed analysis for the precise prediction of viral genome from metagenomes.

This is a good point. We extended and updated the manuscript with the key messages of the benchmarking article by Ho et al. (https://www.biorxiv.org/content/10.1101/2021.04.12.438782v2). WtP executes now by default only the tools that were used in the benchmark study:

“The WtP meta-workflow utilizes several phage identification tools and allows simple execution of a single tool or multiple tools at once. WtP does not favor or disadvantage any prediction tools based on their performance but collects each raw tool output in a user-friendly, easy-to-read result overview.
We did not additionally benchmark the tools integrated into our workflow because the performance of most of them has recently been benchmarked independently [26].
Ho et al. tested Virfinder v1.0, MetaPhinder, PPR_Meta v1.1, Seeker v1.0.3, Virfinder v1.1, VirSorter v1.06, VirSorter2 v2.2.3 and utilized near identical commands, parameters and databases across the benchmarked prediction tools as WtP. Another benchmark would thus only duplicate prior work.
Most tools performed well in the benchmarking of Ho et al., detecting the majority of phage sequences while keeping false positives low. PPR-Meta and VirSorter2, which use two different machine learning methods, had the best performance across the tools.

However, k-mer tools outperformed reference similarity and gene-based tools when tested on positive and negative phage datasets. Tests with randomly shuffled phage sequences showed a clear difference between machine learning and conventional tools.
The performance of most tools dropped significantly when a real metagenomic dataset was used compared to the RefSeq benchmark. The k-mer prediction tools showed a smaller drop in F1-score based on their RefSeq benchmark compared to reference similarity/gene-based tools as described by Ho et al. [26].
The group also pointed out that the tools with machine/deep learning algorithms can detect novel phages. However, their performance may be unpredictable when exposed to novel data with features that differ from those used in the training sets.
Therefore, we believe that a combination of phage prediction algorithms (machine/deep learning and similarity/gene-based) is a good compromise for unknown and novel datasets.
WtP deploys the benchmarked tools by default. If users wish to deploy other tools that were not benchmarked by Ho et al., they can activate them easily.
While a sensical approach, multiple tools can be combined in the prediction step to yield an "ensemble" approach. To benchmark this approach, however, against individual tools is beyond the scope of this work, which aimed to facilitate the accessibility to phage prediction tools."

We agree with the reviewer that the results may confuse the user, and that is why we added information (WtP v1.2.0) to the result report , in the end, to help understand the data.

Also, I feel that, because the development of bioinformatics tools is quite rapid, integrated platforms like WtP will be outdated very soon without continuous effort for maintenance and upgrade to assimilate future novel tools. I understand the 'sustainability' of the tool is out of the journal scope, but the perspective on this point will be better to be described in the manuscript or GitHub page.

We understand the concerns, and that is why we choose nextflow, which heavily improves continuous integration. The modular code makes it very easy to include new tools or integrate tools if the users request them. Additionally, as you can directly see in the code at Github we already had some developers from popular phage prediction tools that were eager to help with implementation steps (Phigaro). Further, we went with individual containerization (docker/singularity) for each integrated bioinformatic software to avoid any typical incompatibility issues and installation problems. (containerized nextflow workflows are rather "stable" throughout the years). Therefore, we designed the workflow to be as future-proof as possible and as easy to main as possible. We also considered good coding practice and code readability aspects.
We aim to update WtP regularly (similar to poreCov: https://github.com/replikation/poreCov) while responding to issues (e.g. see our git issue tab)
(https://github.com/replikation/What_the_Phage/issues;
https://github.com/replikation/What_the_Phage/issues?q=is%3Aissue+is%3Aclosed).
In the next few months, we will write a tutorial on how to integrate new tools, so developers do not rely completely on us.

Some tools (e.g., Virsorter2) can be used to predict viruses out from common bacteriophages, e.g., NCLDV and virophage (See the original article of VirSorter2). Those kinds of viruses should be described briefly in this section as well as common dsDNA phages.

Thank you for the suggestion. We updated the information in our manuscript:

"The dsDNA-tailed phages, possibly make up the majority of phages on the planet [3]. Single-stranded DNA (ssDNA), single-stranded RNA (ssRNA), and double-stranded RNA viruses are minor groups [4]."

Assembly-free long read is described here, but I think this is a bit far from the scope of this manuscript. Indeed, the dataset used in this study (ERR575692) is derived from Illumina HiSeq and the performance of assembly-free long-read dataset was not analyzed in this study. I think the descriptions could be moved to the Discussion section rather than the Introduction. Rather than that, it would be better to add more attractive descriptions about studies of phage genomes identified from short-read metagenomes to emphasize the importance of phage prediction and the value of the proposed tool, WtP. e.g., History of viral genomics using metagenomic dataset, recent technical improvement of metagenomics, phylogenetic diversity of phages, discovery of novel and uncharacterized phages from environmental metagenome, etc.

Thank you for the suggestion. We agree that it is far from the scope of our manuscript. The sentence was intended to show that with long-read sequencing technologies, especially with the MinION device, everyone can sequence phages everywhere (on site) and therefore find more phages in the future. We clarified this in the introduction and data description section:

“It should be noted that due to the genome size of phages ranging from 5 kbp to 500 kbp [10], their entire genome can be sequenced via long-read technologies (e.g., Oxford Nanopore Technologies or PacBio) [11]. These sequencing techniques facilitate phage genome recovery in their natural habitat without the need to culture their hosts to isolate the phages [2] and sequencing of soil or ocean samples on-site, e. g., with the portable MinION sequencing device. Such technological developments led to a rapid increase in human gut virome studies [12] and the discovery of novel, uncharacterized phages from environmental metagenomes [13,14].”

and:

“The dataset analyzed in this study (ERR575692) is derived from Illumina HiSeq data”

Only 5 out of 11 tools that used in WtP were introduced here. The remaining 6 tools would be better to also cite here with a brief explanation of those strategies for virus prediction.

Thank you for the suggestion. We updated the information in our manuscript:

“Existing prediction tools rely on direct comparison of sequence similarity [15,16], sequence composition [17,18], and models based on these features derived through learning algorithms [15,16,19]. The phage prediction tool DeepVirFinder uses a k-mer-based deep learning method using convolutional neural networks and builds on its predecessor VirFinder [18,20]. PPR-Meta also utilizes convolutional neural networks to identify both phages and plasmids [19]. Metaphinder integrates BLAST hits to multiple genomes in a database to identify phage sequences in assembled contigs [21]. Seeker and VirNet work with a deep learning framework that uses long short-term memory models that do not depend on sequence motives [22,23], while Vibrant utilizes deep learning neural networks based on protein signatures [15]. Virsorter2 builds on the strategy of Virsorter (first iteration) by applying machine learning to evaluate the viral content using genomic features [16,24]. Phigaro uses pre-computed sets of pVOG profile HMMs (Hidden Markow Models) [25]”

Also, MARVEL was cited here but not used in WtP.

Thank you for the suggestion. We removed MARVEL-related information in our manuscript.

Figure 1 is different from the one on the GitHub page (https://mult1fractal.github.io/wtp-documentation/figures/wtp-flowchart-simple.png), which seem to be better than the Figure 1.
What 'DAG' means?

Thank you for the suggestion. We updated the figure and caption in our manuscript:

“Figure 1: Simplified “What the Phage” workflow Flowchart. Sequence input (yellow) can either be first-run through the “prediction” and subsequently “Annotation & Taxonomy” as a whole or used directly as an input for the “Annotation & Taxonomy” only. Each of the multiple phage prediction tools can be individually controlled if needed (tool control).”

To clarify: DAG stands for direct acyclic graph and is a Flowchart of the processes and tools WtP utilizes.

'a metagenome assembly' could rephrase like 'metagenomic assembled contigs'

Thank you for the suggestion. We changed the information to:

“The first stage takes a multi-fasta file as input (e.g., a metagenomic assembled contigs), formats it to the demands of each tool, and filters sequences below a user-defined length threshold (1,500 bp by default) via SeqKit v0.10.1 [28]”

Metaphinder and Seeker are here with 'no release version'. I understand the situation but I feel this description is not good for reproducing the analysis. To specify the version of tools even if lack the official release version, mention the last commit date (For Metaphinder, Aug 10, 2021) or GitHub commit ID (bebc447d00ec9ff9f4960f38b627d8651262ff72) is likely a good way.

Thank you for the suggestion. The tool container that we used are already “locked in” on a specific commit id, but we completely agree that its important information for the manuscript:

“The phage prediction process is performed by eleven different tools (14 approaches) in parallel: VirFinder v1.1 [18], PPR-Meta v1.1 [19], VirSorter v1.0.6 (with and without virome mode) [16], DeepVirFinder v1.0 [20], Metaphinder with no release version (using default database and own database (Zheng et al. database, Github commit ID bebc447d00ec9ff9f4960f38b627d8651262ff72) [21], sourmash v2.0.1 [17], Vibrant v1.2.1 (with and without virome mode) [15], VirNet v0.1 [23] Phigaro v2.2.6 [25], Virsorter2 v2.0 [24] and Seeker [22] with no release version (Github commit ID 9ae14887dcd4295f4340626d06d8848cead2102d).”

In this manuscript, Prodigal was used for gene prediction. However, accurate gene prediction from phage genome is still difficult (see https://academic.oup.com/bioinformatics/article/35/22/4537/5480131). This fact have been affect both the phage prediction and functional gene annotation in the field of virology. I think the difficulty of gene prediction from phage genome and potential room for improvement should be noted in the discussion section.

Thank you for the suggestion. We added the information to our manuscript in the limitations section.

“Furthermore, WtP uses default database(s) or the original trained model(s) provided with each stand-alone prediction tool. We note that most casual users are unlikely to retrain these tools before their use. Accurate gene prediction from the phage genome is still difficult [41]. This fact has affected both phage prediction and functional gene annotation in virology. New phage gene databases and algorithms could improve the quality of gene prediction in the future. We, therefore, implemented the function to provide, e.g., more recent databases to improve gene annotation.”
We also updated the WtP-code (WtP v1.2.0) so users can provide their own gene annotation databases

The sentence ' ~ IMG/VR, iVirus, or VERVE-NET' here should be with appropriate citations or URLs. I found a paper of iVirus.

Thank you for the suggestion. We updated it in our manuscript:

“All the results are individually filterable so that the user can consider additional insights or information provided by community platforms such as IMG/VR [34] or iVirus [35].”

WTP -> WtP

Thank you for the suggestion. We updated it in our manuscript.

Figure 3. X-axis title of left-bottom bar plot and Y-axis title of top-right bar plot. viral -> phage

Thank you for the suggestion. We updated the figure and the WtP-code:

What 'prediction values' mean? Are these scores generated by each prediction tool?

Thank you for the suggestion. We clarified the meaning of prediction values in our manuscript:

“In general, the prediction values (p-values, scores and outputs generated by the phage prediction tools) are above >0.7 for the first four sequences/contigs (NODE_14, NODE_13, NODE_12, NODE_30), indicating high consensus among the prediction tools, although in some cases tools prediction values were below 0.5 (Phigaro: NODE_30, Seeker: NODE_12 and NODE_30, Virnet: NODE_12 and Virsorter2: NODE_30).”

Figure 4. X-axis texts. Unify the format to either NodeID:assignment (e.g., NODE_5:unknown) or assignment:NodeID (T3:NODE_14).

Thank you for the suggestion. We updated it in our manuscript:

The sequences matched with 100% identity to Salmonella enterica (Salmonella enterica strain FDAARGOS_768 chromosome, complete genome), but not to prophage sequences. ' here. Does the sentence mean that the contig NODE_5 and NODE_8 were mis-predicted as prophage by CheckV?

Thank you for the suggestion. We clarified the part in our manuscript:

“These results were manually confirmed using NCBI’s blastn (nr/nt database). The sequences matched with 100% identity to Salmonella enterica (Salmonella enterica strain FDAARGOS_768 chromosome, complete genome), but not to prophage sequences. Furthermore, NODE_8 had 1.37 times the contig length of the matched reference (from CheckV), and NODE_5 had 3.24 times the contig length of the matched reference, which may have influenced the NCBI blastn search. Salmonella enterica is known to host prophages [40]; therefore, the identified prophage sequences via CheckV are plausible results.”

Table 1. completeness -> completeness (%)

Thank you for the suggestion. We updated it in our manuscript:

Phage name Contig_id Gene count CheckV
quality Completeness
[%] contig length [bp]
unknown1 NODE_5 107 Complete 100.0 114,288
unknown2 NODE_8 71 High-quality 100.0 63,147
VPE25 NODE_6 137 High-quality 99.99 86,514
phiX174 NODE_30 8 Medium-quality 89.35 5,441
T3 NODE_14 43 High-quality 93.34 37,380
T7 NODE_13 53 Complete 99.48 39,820
P22 NODE_12 67 Complete 100.0 41,715

Add citation in the line ' At least one multitool approach was implemented on a smaller scale by Ann C. Gregory et al. (comprising only VirFinder and VirSorter). '

Thank you for the suggestion. We updated it in our manuscript:

“At least one multitool approach was implemented on a smaller scale by Ann C. Gregory et al. (comprising only VirFinder and VirSorter) [29]”

16. Lack doi.
18. Lack doi.

19. Lack doi.

Thank you for the suggestion. We updated the missing doi in our manuscript.

Reviewer #2

In this manuscript, the authors developed an integrated workflow WtP for identification, annotation and taxonomy of phage sequences. Based on Docker and Nextflow, WtP integrates 11 phage sequence identification tools (including 14 approaches), two functional annotation and taxonomy tools (Prodigal and HMMER), and a visualizing tool (chromoMap). When using WtP, it is convenient that users do not need to install each tool and can avoid the conflict between each installation package and between operating systems. Also, the WtP tool was applied to the artificial microbiome. The threshold of each phage sequence prediction tool can be manually adjusted and outputted. Annotation and taxonomy results of phage sequences can be further visualized by CheckV and by chromeMap tool. However, there are some limitations in this manuscript. For the annotation and taxonomy stage, only the Prodigal tool was used for gene prediction, and no other gene prediction tools (especially the phage-specific tools). It is necessary for an integrated workflow to include other similar tools. WtP needs at least 4 GB of memory and 75 GB of storage, so the author should develop a web version or at least a graphical interface version of WtP for its prevalence.

Major comment:

1. Except for sequence identification, host prediction (e.g., HoPhage, PHP, and VirHost Matcher-Net) and lifestyle prediction (e.g., DeepPhage, PhagePred) of phage sequences are also important in microbial communities. However, WtP did not involve those functions.

We agree that host prediction and lifestyle prediction are important parameters to describe phages, and it is correct that WtP does not include these. WtP focuses on the reliable execution of many state-of-the-art phage prediction tools to address the major bottleneck of phage identification.
We wanted to make sure that phage identification is reproducible and installation of the several included tools is not needed, as WtP takes care of all the necessary steps to install, run, and collect the tool outputs in an easy-to-read and interactive result report.
From our own experience, we know how hard it is to get several phage identification tools to run in one environment when there is no bioinformatic knowledge available to help. This is also the general feedback of other users (on conferences, GitHub, and via E-mail) we receive.
We understand the concerns, and that is why we choose nextflow, which heavily improves continuous integration. The modular code makes it very easy to include new tools or integrate tools if the users request them. Further, we went with individual containerization (docker/singularity) for each integrated bioinformatic software to avoid any typical incompatibility issues and installation problems (containerized nextflow workflows are rather “stable” throughout the years). Therefore, we designed the workflow to be as future-proof as possible and as easy to maintain as possible. We also considered good coding practice and code readability aspects. We aim to update WtP regularly (similar to poreCov: https://github.com/replikation/poreCov) while responding to issues (e.g. see our git issue tab) (https://github.com/replikation/What_the_Phage/issues; https://github.com/replikation/What_the_Phage/issues?q=is%3Aissue+is%3Aclosed).
However, good coding practice and workflow design are best when focused on one problem/topic at a time. Having dedicated workflows for distinct tasks e.g. a workflow for phage prediction, a workflow for host prediction, and a workflow for lifestyle prediction, is more convenient than managing a bloated and messy workflow that includes all phage-related tools and is hard to maintain, unlike WtP.
A good compromise could be to develop a new workflow with two - three phage prediction tools, a host prediction tool, and a lifestyle prediction tool in cooperation with international phage experts.

2. In addition to the web version or graphical interface version of WtP, the author can also consider a video demo or usage illustration. To clarify the purpose of this study, I think it would be better to add the phrase 'a web server of ...' or 'a GUI platform of ...' into the title.

This would require a lot of additional and continuous funding for computer hardware and maintenance. Something like this is only possible for small-scale tools. Therefore, nearly never done for any compute-intensive bioinformatic tool or workflow. However, there is a reason we and many others are implementing workflows in nextflow as one could execute (every) nextflow workflow, such as WtP, directly via the "Tower." (https://cloud.tower.nf/). The developer of nextflow provides a "universal"

GUI/Dashboard to execute nextflow workflows. Nextflow tower offers free and paid tiers and a simplistic GUI. So it's up to each user if they, e.g., want to execute WtP via a Dashboard such as Tower. There are many tutorials for this available; it basically comes down to adding the workflow you want to use and providing the compute environment (where to run, e.g., google cloud, AWS, etc.). Web hosting servers usually have a limited lifespan until they expire. Some web tools need a lot of time to upload big datasets. On the contrary, WtP can preload all necessary databases and then execute WtP without being connected to the internet at all, which is especially useful when having bad internet connections (https://github.com/replikation/What_the_Phage/issues/167).

3. In 'Analysis' Section (Page 12), only four contigs of phage sequences can be annotated in artificial data: P22 (NODE_12), T3 (NODE_14), T7 (NODE_13) and phiX174 (NODE_30). The 'predicted_organism_name' of the remaining 102 phage contigs are 'no match found'. Can WtP improve or add more databases to annotate more contigs?

We understand your concern. We described in the data description section that the artificial metagenome consists of germ-free mice feces bacteria and phage cultures:

“Kleiner et al. [38] generated an artificial microbiome via bacteria and phage cultures in mice feces (germ-free C57BL/6 J mice) and sequenced the sample. The group added six phages: P22, T3, T7, ϕ6, M13, and ϕVPE25, and two bacteria (Listeria monocytogenes and Bacteroides thetaiotaomicron) to germ-free feces. We, therefore, expect the prediction of the six known phages and possibly other phage sequences related to both bacteria strains. Still, false-positive hits and tool disagreements are plausible results during the phage prediction process.‟

WtP identified all relevant phages that were also identified by Kleiner et al. . Therefore, the remaining contigs could be of Bacteria or mouse origin.
Your suggestion to add more databases for phage annotation is very good. We updated the WtP workflow (v1.2.0) so that users can provide their own annotation databases:

“Furthermore, WtP uses default database(s) or the original trained model(s) provided with each stand-alone prediction tool. We note that most casual users are unlikely to retrain these tools before their use. Accurate gene prediction from the phage genome is still difficult [41]. This fact has affected both phage prediction and functional gene annotation in virology. New phage gene databases and algorithms could improve the quality of gene prediction in the future. We, therefore, implemented the function to provide, e.g., more recent databases to improve gene annotation.”

4. In 'Analysis' Section (Page 14), the author mentions 'No specialized phage assembly strategy or any cleanup step was included during the assembly step'. I think it is unreasonable, and the downstream analysis will inevitably be affected by the impurity sequences.

We understand the reviewer's concerns. However, as the organisms of this dataset were known beforehand, we were able to retrieve all phages that the group of Kleiner et al. found in their analysis and also the internal Illumina control. We, therefore, think a specialized phage assembly strategy or any cleanup step was not necessary in this special case. To get rid of potential impurity, WtP filters and analyzes only sequences above 1500 bp by default (this value is adjustable).
We agree with the reviewer that if users want to analyze unknown datasets from sequencing data, phage assembly strategies (especially for Illumina sequenced datasets) are necessary to improve results.

5. In Figure 2, it is possible to export results in the form of 'csv', 'pdf' or 'excel'. Can WtP export all the predicted phage sequences in the form of 'fasta'. The author should describe how to change or add the database during the annotation and classification phases.

Yes, it is possible. In the report, above the tables, there are buttons for exporting the data in Excel, CSV, or PDF files. For better understanding, we added the information in the figure 2 caption:

“Figure 2: Example figure of the “Phage prediction by contig table”-section of the result report

summarizes the tool outputs for the analyzed sample ERR575692. The full result report can be found here: https://replikation.github.io/What_the_Phage/. All tables can be exported as Excel, PDF, or CSV files by using the buttons above the tables."

Your suggestion to add more databases for phage annotation is very good. We updated the WtP workflow (v1.2.0) so that users can provide their own annotation databases:

"Furthermore, WtP uses default database(s) or the original trained model(s) provided with each stand-alone prediction tool. We note that most casual users are unlikely to retrain these tools before their use. Accurate gene prediction from the phage genome is still difficult [41]. This fact has affected both phage prediction and functional gene annotation in virology. New phage gene databases and algorithms could improve the quality of gene prediction in the future. We, therefore, implemented the function to provide, e.g., more recent databases to improve gene annotation."

Can WtP export all the predicted phage sequences in the form of 'fasta'.

We understand this request. WtP collects the outputs of the phage prediction tools solely. The problem: How to set the cut-off if a sequence is a phage or not. Potential false negative or false positive sequences could be exported. Therefore we think the user should further analyze the results provided and extract the sequences of interest by themselves using the provided tutorial in the result report:

"Extract contigs of interest

```
# Filter the Phage prediction by contig table to your liking
# Click on the CSV-Button (this will download the Phage prediction by contig table)
# Open your Linux-Terminal
mkdir contigs_of_interest
cd contigs_of_interest
# Copy the downloaded Phage prediction by contig table to the contig_IDs_of_interest -folder
# Copy the input_fasta to the contig_IDs_of_interest -folder
cp WtP_results/your_sample/Input_fasta/your_input_fasta.fa.gz /foo/bar/contigs_of_interest
# Get contig IDs of interest
tail -n+2 final_report.utf8.csv | tr -d '"' | cut -f2 -d"," > contig_IDs_of_interest.txt
# via Docker: use Seqkit to extract contigs of interest of your input fasta-file
docker run --rm -it -v $PWD:/input nanozoo/seqkit:0.13.2--cd66104
cd input
seqkit grep --pattern-file contig_IDs_of_interest.txt your_input_fasta.fa.gz > contigs_of_interest.fa
# Finally, close the docker with ctrl + d"
```

"We think it is still the user's task to select the most likely phage contigs, extract them, and use them for a more detailed and curated analysis, based on the phage prediction tool outputs, collected in the result report. WtP can only provide an all-encompassing starting point for phage."

Minor comment:

1. In 'Functional annotation & Taxonomy' Section (Page 8), 'Figure 3' in the sentence 'All annotations are summarized in an interactive HTML file via chromoMap (see Figure 3)' should be 'Figure 4'.

Thank you for the suggestion. We updated it in our manuscript according to your suggestion:

"All annotations are summarized in an interactive HTML file via chromoMap [33] (see Figure 4)"

2. The column of 'Computeness' in Table 1 missed the unit, and the author could add an outer border to Table 1.

Thank you for the suggestion. We updated it in our manuscript according to your suggestion:

Phage name Contig_id Gene count CheckV
quality Completeness
[%] contig length [bp]

unknown1 NODE_5 107 Complete 100.0 114,288
unknown2 NODE_8 71 High-quality 100.0 63,147
VPE25 NODE_6 137 High-quality 99.99 86,514
phiX174 NODE_30 8 Medium-quality 89.35 5,441
T3 NODE_14 43 High-quality 93.34 37,380
T7 NODE_13 53 Complete 99.48 39,820
P22 NODE_12 67 Complete 100.0 41,715

3. Figure 2 need to be clearer.

Thank you for the suggestion. We updated it in our manuscript according to your suggestion. We clarified what the reader sees in the figures.

"Figure 2: Example figure of the "Phage prediction by contig table"-section of the result report. The "Phage prediction by contig table"-section summarizes the tool outputs for the analyzed sample ERR575692. The full result report can be found here: https://replikation.github.io/What_the_Phage/. All tables can be exported as Excel, PDF, or CSV files by using the buttons above the tables."

3. Figure 3 need to be clearer.
Thank you for the suggestion. We updated the figure. We also simplified the description and how to read this plot as much as possible. How to read the diagram: For example, 53 phage contigs are found by six tools (DeepVirFinder, Metaphinder-own-DB, Metaphinder, PPRmeta, seeker, and Virfinder). Another 42 non-overlapping phage contigs are found by these tools but also virnet.
We chose the UpSet diagram as it is clearer for visualizing the intersection between more than 3 sets (phage prediction tools) in comparison to standard Venn diagrams.
If the reviewer could provide a better and simpler description of this diagram, we would be happy to include this in our manuscript.

Figure 3: UpSet plot summarizing the prediction performance of each tool for the sample ERR575692. The total amount of identified phage contigs per tool is shown in blue bars on the left. Black, vertical bars visualize the number of contigs that each tool or tool combination has uniquely identified. Each tool combination is shown below the vertical barplot as a dot matrix. How to read the diagram: For example, 53 phage contigs are found by six tools (DeepVirFinder, Metaphinder-own-DB, Metaphinder, PPRmeta, Seeker, and VirFinder). Another 42 contigs are found by these tools but also virnet.

4. Page 5. 'approach to gain' should be 'approach to gaining'.

Thank you for the suggestion. We updated it in our manuscript according to your suggestion:

"Sequencing the entire DNA of environmental samples (metagenomics) is an essential approach to gaining insights into the microbiome and functional properties."

5. Page 13. 'In addition to' should be 'In addition to'.

Thank you for the suggestion. We updated it in our manuscript according to your suggestion:

"In addition to the phages mentioned above, two more large contigs with capsid and tail gene annotations indicate prophage(s) of Salmonella enterica (contig NODE_5 and NODE_8)."

Close
