# Supplementary material for: What the Phage: a scalable workflow for the identification and analysis of phage sequences
Source: Gigascience. 2022 Nov 18;11:giac110. doi: 10.1093/gigascience/giac110 (PMC9673492; doi:10.1093/gigascience/giac110)
Supplement: giac110_Reviewer_1_Report_Original_Submission — Satoshi Hiraoka -- 6/20/2022 Reviewed [file giac110_reviewer_1_report_original_submission.pdf]

Reviewer Report

Title: What the Phage: A scalable workflow for the identification and analysis of phage sequences

Version: Original Submission **Date:** 6/20/2022

Reviewer name: Satoshi Hiraoka

Reviewer Comments to Author:

In this manuscript, the authors developed a new tool, What the Phage (WtP), for comparison of the output from multiple bioinformatics tools to predict phage sequences from genomic or metagenomic datasets. The purpose of this study is some or less meaningful. As the authors described in the Introduction section, currently it is difficult to predict reliable viral genomes, especially from culture-independent metagenomic datasets precisely because of the lack of knowledge about viral genomes in current protein/genome databases. There are many bioinformatics tools already proposed and some of them are widely used in microbiology, however, the outputs from these tools are frequently varied and conflicted among them. However, there is no good integrative platform to compare the outputs. Here, the proposed tool easily generates well-summarized output derived from multiple tools, and thus, the tool might be facilitated the analysis of phage prediction in the field of microbiology. Indeed, the authors conducted (only but) one case study using real phage genomes and reported reasonable performance. I feel the tool has some potential to contribute to the wide fields of viral genomics. However, the user of this tool should keep in mind the fact that the tool just summarizes the output of multiple phage-prediction tools, meaning does not evaluate the reliability of the output, as described in the Discussion section. I feel thus the tool sometimes may lead to misunderstandings or make the users confuse rather than help them. It should emphasize that the majority decision among the multiple tools does not always bring the best result. The users may need further detailed analysis for the precise prediction of viral genome from metagenomes.
Also, I feel that, because the development of bioinformatics tools is quite rapid, integrated platforms like WtP will be outdated very soon without continuous effort for maintenance and upgrade to assimilate future novel tools. I understand the 'sustainability' of the tool is out of the journal scope, but the perspective on this point will be better to be described in the manuscript or GitHub page.
I have some suggestions that would increase the clarity and impact of this manuscript if addressed.
[Background]
Some tools (e.g., Virsorter2) can be used to predict viruses out from common bacteriophages, e.g., NCLDV and virophage (See the original article of VirSorter2). Those kinds of viruses should be described briefly in this section as well as common dsDNA phages.
Assembly-free long read is described here, but I think this is a bit far from the scope of this manuscript. Indeed, the dataset used in this study (ERR575692) is derived from Illumina HiSeq and the performance of assembly-free long-read dataset was not analyzed in this study. I think the descriptions could be moved to the Discussion section rather than the Introduction. Rather than that, it would be better to add more attractive descriptions about studies of phage genomes identified from short-read metagenomes to emphasize the importance of phage prediction and the value of the proposed tool,

WtP. e.g., History of viral genomics using metagenomic dataset, recent technical improvement of metagenomics, phylogenetic diversity of phages, discovery of novel phage lineages from environmental metagenome, etc.
Only 5 out of 11 tools that used in WtP were introduced here. The remaining 6 tools would be better to also cite here with a brief explanation of those strategies for virus prediction. Also, MARVEL was cited here but not used in WtP.
[Design and Implementation]
Figure 1 is different from the one on the GitHub page (https://mult1fractal.github.io/wtp-documentation/figures/wtp-flowchart-simple.png), which seem to be better than the Figure 1.
What 'DAG' means?
[Prediction and Visualization]
'a metagenome assembly' could rephrase like 'metagenomic assembled contigs'
Metaphinder and Seeker are here with 'no release version'. I understand the situation but I feel this description is not good for reproducing the analysis. To specify the version of tools even if lack the official release version, mention the last commit date (For Metaphinder, Aug 10, 2021) or GitHub commit ID (bebc447d00ec9ff9f4960f38b627d8651262ff72) is likely a good way.
[Functional annotation & Taxonomy]
In this manuscript, Prodigal was used for gene prediction. However, accurate gene prediction from phage genome is still difficult (see https://academic.oup.com/bioinformatics/article/35/22/4537/5480131). This fact have been affect both the phage prediction and functional gene annotation in the field of virology. I think the difficulty of gene prediction from phage genome and potential room for improvement should be noted in the discussion section.
[Result report]
The sentence ' ~ IMG/VR, iVirus, or VERVE-NET' here should be with appropriate citations or URLs. I found a paper of iVirus: https://www.nature.com/articles/s43705-021-00083-3
[Other features]
WTP -> WtP
[Analysis]
Figure 3. X-axis title of left-bottom bar plot and Y-axis title of top-right bar plot. viral -> phage
What 'prediction values' mean? Are these scores generated by each prediction tool?
Figure 4. X-axis texts. Unify the format to either NodeID:assignment (e.g., NODE_5:unknown) or assignment:NodeID (T3:NODE_14).
' The sequences matched with 100% identity to Salmonella enterica (Salmonella enterica strain FDAARGOS_768 chromosome, complete genome), but not to prophage sequences. ' here. Does the sentence mean that the contig NODE_5 and NODE_8 were mis-predicted as prophage by CheckV?
Table 1. completeness -> completeness (%)
[Discussion & potential implications]
Add citation in the line ' At least one multitool approach was implemented on a smaller scale by Ann C. Gregory et al. (comprising only VirFinder and VirSorter). '
[References]
16. Lack doi.

18. Lack doi.
19. Lack doi.

Level of Interest

Please indicate how interesting you found the manuscript: Choose an item.

Quality of Written English

Please indicate the quality of language in the manuscript: Choose an item.

Declaration of Competing Interests

Please complete a declaration of competing interests, considering the following questions:

- Have you in the past five years received reimbursements, fees, funding, or salary from an organisation that may in any way gain or lose financially from the publication of this manuscript, either now or in the future?
- Do you hold any stocks or shares in an organisation that may in any way gain or lose financially from the publication of this manuscript, either now or in the future?
- Do you hold or are you currently applying for any patents relating to the content of the manuscript?
- Have you received reimbursements, fees, funding, or salary from an organization that holds or has applied for patents relating to the content of the manuscript?
- Do you have any other financial competing interests?
- Do you have any non-financial competing interests in relation to this paper?

If you can answer no to all of the above, write 'I declare that I have no competing interests' below. If your reply is yes to any, please give details below.

I declare that I have no competing interests

I agree to the open peer review policy of the journal. I understand that my name will be included on my report to the authors and, if the manuscript is accepted for publication, my named report including any attachments I upload will be posted on the website along with the authors' responses. I agree for my report to be made available under an Open Access Creative Commons CC-BY license (http://creativecommons.org/licenses/by/4.0/). I understand that any comments which I do not wish to be included in my named report can be included as confidential comments to the editors, which will not be published.

Choose an item.

To further support our reviewers, we have joined with Publons, where you can gain additional credit to further highlight your hard work (see: https://publons.com/journal/530/gigascience). On publication of this paper, your review will be automatically added to Publons, you can then choose whether or not to claim your Publons credit. I understand this statement.

Yes Choose an item.
