# Supplementary material for: What the Phage: a scalable workflow for the identification and analysis of phage sequences
Source: Gigascience. 2022 Nov 18;11:giac110. doi: 10.1093/gigascience/giac110 (PMC9673492; doi:10.1093/gigascience/giac110)
Supplement: giac110_Reviewer_2_Report_Original_Submission — Huaiqiu Zhu -- 7/7/2022 Reviewed [file giac110_reviewer_2_report_original_submission.pdf]

Reviewer Report

Title: What the Phage: A scalable workflow for the identification and analysis of phage sequences

Version: Original Submission **Date:** 7/7/2022

Reviewer name: Huaiqiu Zhu

Reviewer Comments to Author:

In this manuscript, the authors developed an integrated workflow WtP for identification, annotation and taxonomy of phage sequences. Based on Docker and Nextflow, WtP integrates 11 phage sequence identification tools (including 14 approaches), two functional annotation and taxonomy tools (Prodigal and HMMER), and a visualizing tool (chromoMap). When using WtP, it is convenient that users do not need to install each tool and can avoid the conflict between each installation package and between operating systems. Also, the WtP tool was applied to the artificial microbiome. The threshold of each phage sequence prediction tool can be manually adjusted and outputted. Annotation and taxonomy results of phage sequences can be further visualized by CheckV and by chromeMap tool. However, there are some limitations in this manuscript. For the annotation and taxonomy stage, only the Prodigal tool was used for gene prediction, and no other gene prediction tools (especially the phage-specific tools). It is necessary for an integrated workflow to include other similar tools. WtP needs at least 4 GB of memory and 75 GB of storage, so the author should develop a web version or at least a graphical interface version of WtP for its prevalence.
Major comment:
1. Except for sequence identification, host prediction (e.g., HoPhage, PHP, and VirHost Matcher-Net) and lifestyle prediction (e.g., DeepPhage, PhagePred) of phage sequences are also important in microbial communities. However, WtP did not involve those functions.
2. In addition to the web version or graphical interface version of WtP, the author can also consider a video demo or usage illustration. To clarify the purpose of this study, I think it would be better to add the phrase 'a web server of ...' or 'a GUI platform of ...' into the title.
3. In 'Analysis' Section (Page 12), only four contigs of phage sequences can be annotated in artificial data: P22 (NODE_12), T3 (NODE_14), T7 (NODE_13) and phiX174 (NODE_30). The 'predicted_organism_name' of the remaining 102 phage contigs are 'no match found'. Can WtP improve or add more databases to annotate more contigs?
4. In 'Analysis' Section (Page 14), the author mentions 'No specialized phage assembly strategy or any cleanup step was included during the assembly step'. I think it is unreasonable, and the downstream analysis will inevitably be affected by the impurity sequences.
5. In Figure 2, it is possible to export results in the form of 'csv', 'pdf' or 'excel'. Can WtP export all the predicted phage sequences in the form of 'fasta'.
The author should describe how to change or add the database during the annotation and classification phases.
Minor comment:
1. In 'Functional annotation & Taxonomy' Section (Page 8), 'Figure 3' in the sentence 'All

annotations are summarized in an interactive HTML file via chromoMap (see Figure 3)' should be 'Figure 4'.
2. The column of 'Computeness' in Table 1 missed the unit, and the author could add an outer border to Table 1.
3. Figure 2 and Figure 3 need to be clearer.
4. Page 5. 'approach to gain' should be 'approach to gaining'.
5. Page 13. 'In addition to' should be 'In addition to'.

Level of Interest

Please indicate how interesting you found the manuscript: Choose an item.

Quality of Written English

Please indicate the quality of language in the manuscript: Choose an item.

Declaration of Competing Interests

Please complete a declaration of competing interests, considering the following questions:

- Have you in the past five years received reimbursements, fees, funding, or salary from an organisation that may in any way gain or lose financially from the publication of this manuscript, either now or in the future?
- Do you hold any stocks or shares in an organisation that may in any way gain or lose financially from the publication of this manuscript, either now or in the future?
- Do you hold or are you currently applying for any patents relating to the content of the manuscript?
- Have you received reimbursements, fees, funding, or salary from an organization that holds or has applied for patents relating to the content of the manuscript?
- Do you have any other financial competing interests?
- Do you have any non-financial competing interests in relation to this paper?

If you can answer no to all of the above, write 'I declare that I have no competing interests' below. If your reply is yes to any, please give details below.

I declare that I have no competing interests.

I agree to the open peer review policy of the journal. I understand that my name will be included on my report to the authors and, if the manuscript is accepted for publication, my named report including any attachments I upload will be posted on the website along with the authors' responses. I agree for my report to be made available under an Open Access Creative Commons CC-BY license (http://creativecommons.org/licenses/by/4.0/). I understand that any comments which I do not wish to

be included in my named report can be included as confidential comments to the editors, which will not be published.

Choose an item.

To further support our reviewers, we have joined with Publons, where you can gain additional credit to further highlight your hard work (see: https://publons.com/journal/530/gigascience). On publication of this paper, your review will be automatically added to Publons, you can then choose whether or not to claim your Publons credit. I understand this statement.

Yes Choose an item.
